# Immunoinformatics and Molecular Docking Studies Predicted Potential Multiepitope-Based Peptide Vaccine and Novel Compounds against Novel SARS-CoV-2 through Virtual Screening

**DOI:** 10.1155/2021/1596834

**Published:** 2021-02-26

**Authors:** Muhammad Waqas, Ali Haider, Abdur Rehman, Muhammad Qasim, Ahitsham Umar, Muhammad Sufyan, Hafiza Nisha Akram, Asif Mir, Roha Razzaq, Danish Rasool, Rana Adnan Tahir, Sheikh Arslan Sehgal

**Affiliations:** ^1^Department of Bioinformatics and Biotechnology, Government College University, Faisalabad, Pakistan; ^2^Department of Environmental Sciences, Quaid-e-Azam University, Islamabad, Pakistan; ^3^Department of Biological Sciences, International Islamic University, Islamabad, Pakistan; ^4^Department of Biosciences, COMSATS University, Sahiwal Campus, Islamabad, Pakistan; ^5^Department of Bioinformatics, University of Okara, Okara, Pakistan

## Abstract

**Background:**

Coronaviruses (CoVs) are enveloped positive-strand RNA viruses which have club-like spikes at the surface with a unique replication process. Coronaviruses are categorized as major pathogenic viruses causing a variety of diseases in birds and mammals including humans (lethal respiratory dysfunctions). Nowadays, a new strain of coronaviruses is identified and named as SARS-CoV-2. Multiple cases of SARS-CoV-2 attacks are being reported all over the world. SARS-CoV-2 showed high death rate; however, no specific treatment is available against SARS-CoV-2.

**Methods:**

In the current study, immunoinformatics approaches were employed to predict the antigenic epitopes against SARS-CoV-2 for the development of the coronavirus vaccine. Cytotoxic T-lymphocyte and B-cell epitopes were predicted for SARS-CoV-2 coronavirus protein. Multiple sequence alignment of three genomes (SARS-CoV, MERS-CoV, and SARS-CoV-2) was used to conserved binding domain analysis.

**Results:**

The docking complexes of 4 CTL epitopes with antigenic sites were analyzed followed by binding affinity and binding interaction analyses of top-ranked predicted peptides with MHC-I HLA molecule. The molecular docking (Food and Drug Regulatory Authority library) was performed, and four compounds exhibiting least binding energy were identified. The designed epitopes lead to the molecular docking against MHC-I, and interactional analyses of the selected docked complexes were investigated. In conclusion, four CTL epitopes (GTDLEGNFY, TVNVLAWLY, GSVGFNIDY, and QTFSVLACY) and four FDA-scrutinized compounds exhibited potential targets as peptide vaccines and potential biomolecules against deadly SARS-CoV-2, respectively. A multiepitope vaccine was also designed from different epitopes of coronavirus proteins joined by linkers and led by an adjuvant.

**Conclusion:**

Our investigations predicted epitopes and the reported molecules that may have the potential to inhibit the SARS-CoV-2 virus. These findings can be a step towards the development of a peptide-based vaccine or natural compound drug target against SARS-CoV-2.

## 1. Background

There are a variety of human diseases with unknown etiology. A viral parentage has been purposed for numerous diseases and also has significance to search new viruses [1]. Various difficulties have been faced which scrutinize new viruses, such as some viruses do not replicate *in vitro* and have cytopathic effects (CPE). The viruses that are unable to replicate *in vitro* leads to the failure of virus discovery. The DNA-amplified restriction fragment length polymorphism (cDNA-AFLP 4) technique helps to identify the new viruses including the discovery of new coronavirus [1].

Coronaviruses, a genus of the Coronaviridae family, are enveloped viruses recognized as of large plus RNA strand genome. The size of RNA is 27-32 kb and polyadenylated. There are three groups of coronaviruses that are serologically distinct. Viruses are characterized within each group by their genomic sequence and host range [2]. Coronaviruses have been discovered in mice, turkeys, cats, horse, and humans and cause many diseases including respiratory tract and gastroenteritis [2].

Two human viruses (HCoV-229E, HCoV-OC43) were identified in the mid-1960s and are known to cause the common cold. The recently identified SARS-CoV can cause a life-threatening pneumonia and is the most pathogenic human coronaviruses identified thus far [3]. SARS-CoV is probable to occupy in animal source and recently initiated the epidemic in humans through zoonotic transmission [4]. SARS-CoV is the first membrane of a fourth group of coronaviruses [5].

In Wuhan (Hubei province, China), multiple patients associated to Hunan south China seafood market diagnosed with third zoonotic human coronavirus (CoV) of the century emerged in 31st of December 2019. CoV is similar to severe acute respiratory syndrome coronavirus (SARS-CoV) and Middle East respiratory syndrome coronavirus (MERS-CoV) infections including fever, lung infiltration, and difficulty breathing [6]. After an extensive speculation about the causative agent of CoV, the identification of novel CoV was announced by the Chinese Center for Disease Control (CDS) on 19th of January 2020 [7]. The novel CoV, SARS-CoV-2, was insulated from a single patient and later corroborated from 16 more patients [8]. The viral pneumonia of SARS-CoV-2 was quickly predicted as the likely causative agent, while not yet confirmed.

The first sequence of SARS-CoV-2 has been submitted after its conformation [9]. Later, five more sequences of SARS-CoV-2 were deposited to the GSAID database on 11th of January from Chinese institutes [10] (Supplementary 1); multiple sequence alignment of SARS-CoV, MERS-CoV, and SARS-CoV-2 carried out and conserved part in DNA, as well as protein sequence, was observed. Hundreds of human deaths were linked with infection having significant morbidities with the age>50. Various clinical symptoms have been highlighted such as dry cough, leukopenia, fever, and shortness of breath. The extracorporeal membrane oxygenation of the patients considered severe cases and need supportive care. The infection of SARS-CoV-2 in elderly patients are less virulent as compared to SARS-CoV (10% mortality) and MERS-CoV (35% mortality) [11].

### 1.1. Origin

The source of the SARS-CoV-2 is still unclear, although the initial cases have been associated with the Huanan South China Seafood Market. The early patients present in the Market got the virus through either human-to-human transmission or a more widespread animal source [11].

The samples from the infected market showed positive results for the novel coronavirus while no specific animal association has been identified [12]. Through codon analyses, it is suggested that the snakes might be the possible source of the viral infection [13], although the assertion has been disputed by others [14] including possible animal vectors, and the researchers are trying to discover the source of SARS-CoV-2.

Coronavirus was thought to infect humans and bats more effectively as both are more related to Coronavirus lifecycle [15]. It has been evidenced that several bats are capable of infecting human cells without intermediate adaptation [16]. The human serology data shows the association of bat CoV proteins leads to zoonotic transmission of SARS-like bat coronavirus for deadliest out breaks [17]. MERS-CoV is also a zoonotic virus and have the origin from the bats [18]. The zoonotic contacts of camel has been evidenced in primary cases of MERS-CoV [19]. These lessons from SARS and MERS highlight the importance of rapidly finding source for SARS-CoV-2 in order to stem the ongoing outbreak [19].

### 1.2. Susceptible Populations

With low patient data, who may be most sensitive to SARS-CoV-2 is difficult to make robust resolution. Disease severity such as SARS-CoV and MERS-CoV equated strongly to host the condition including biological sex, age, and the overall health [20], and similar findings have been observed in early patients of SARS-CoV-2. The SARS- and MERS-CoV infection leads to increase the severity and death rate in people over the age of 50 years [21]. The observed patients having novel CoV had poor health conditions including diabetes, kidney or heart function issues, and hypertension that make them more susceptible for MERS-CoV outbreak, while diabetes, smoking, cardiovascular disease, hypertension, and other chronic illness have also been observed. In the majority of deaths and corresponding to findings in animal models [22], the results indicate that vigilance is essential for these weak patients following SARS-CoV-2 infection [22].

### 1.3. Insights from the Sequence

Dr. Zhang's group at Fudan University and many other groups in China instance the dedication and increased the capacity of the scientific infrastructures in China by rapid sequencing of nearly 30,000 nucleotide of the (COVID) genome [23]. The whole genome analyses of SARS-CoV-2 showed ~80% nucleotide identity to the original SARS epidemic virus. The two different bat SARS-like CoVs (ZC45 and ZXC21) shared ~89% identity with the genome of SARS-CoV-2 [24]. It has been observed that the novel CoV showed recombination with previously identified bat coronaviruses through phylogenetic analyses [25]. A CoV sequence of bat (RaTG3) having 92% sequence identity with the novel virus supports the bat origins for the SARS-CoV-2 [14].

The SARS-CoV-2 spike protein has roughly 75% amino acid identity with SARS-CoV [26] while the SARS-CoV-2 receptor-binding domain (RBD) is 73% conserved with spike RBD of SARS-CoV by narrowing analysis relative to the epidemic RBD [27]. The receptor-binding domain of SARS-CoV-2 was capable of binding with ACE2 in the context of the SARS-CoV spike protein [28].

### 1.4. Genomic Features and Lifecycle of the Coronavirus

Coronaviruses have unique club-like spikes, and the RNA genome is larger than other virus which leads to a unique mode of replication. Coronaviruses contain ~30 kb of positive-strand RNA genome [29]. The significant features of coronavirus genomes include a 5′ caped end which plays an important role in the replication of RNA, as 5′ end has a leader sequence along with a UTR region, possessing essential loops. The 3′ poly-A tail end has essential structures for RNA genome synthesis and replication [30]. These two modifications allow RNA viruses for translation of replication (replicase) proteins [23].

**Figure 1:**
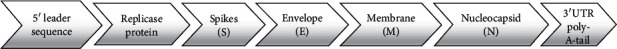
The organization of Coronavirus genome, which contains a 5′ end, a leader sequence, replicase protein (important for replication of whole genome), spikes, envelope, membrane, nucleocapsid, and a 3′UTR poly-A-tail end.

A coronavirus genome has significant parts and helps for the synthesis and replications of whole genome (Figure 1) [31].

The conformed cases of virus have been confirmed by 25 countries [32–34] Tables 1 and 2 (Supplementary 1).

Our current study is aimed at exploring and identifying potential B- and T-cell epitopes through immunoinformatics approaches which help to design effective vaccine against deadly SARS-CoV-2. In addition, the study is aimed at pointing out specific peptides from coronaviral proteome, which have ability to bind with major histocompatibility complex (MHC), one of the most crucial step in vaccine designing. Different bioinformatics tools are applied to follow immunoinformatics approach.

## 2. Methods

### 2.1. SARS-CoV-2 Sequence Retrieval

The primary amino acid sequence of coronavirus protein was extracted from the crystal structure of SARS-CoV-2 main protease in complex with an inhibitor N3 from Protein Data Bank (PDB ID: 6LU7) [35]. The individual sequence length of corona viral protein was 306 amino acids from the genome polyprotein, and a three-dimensional (3D) structure was determined by X-ray diffraction having 2.16 Å resolution. The physiochemical properties of the selected protein were evaluated by using ProtParam [36].

### 2.2. Multiple Sequence Alignment (MSA)

MSA is performed on all three full-length genomes (SARS-CoV = NC_004718, MERS-CoV = NC_019843.3, and SARS-CoV-2 = NC_045512.2), all genomic sequences taken by GenBank [37, 38] and multiple sequence alignment carried out by Clustal Omega [39, 40]. The conserved parts were labeled by using WebLogo3 [41].

### 2.3. Conformational and Linear B-Cell Epitopes Prediction

The interaction of the antigen B-cell epitope with B-lymphocyte classifies the B-lymphocytes to differentiate into the two types of cells as memory cells and antibody-secreting plasma [42]. The accessibility and hydrophilic nature were considered the key features of the B-cell [43] by accessing the immune epitope database and analysis resource (IEDB) (http://www.iedb.org/) as stated by flexibility prediction of Karplus and Schulz [44], hydrophilicity prediction of Parker et al. [43], antigenicity scale of Kolaskar and Tongaonkar [45], and Emini et al. surface accessibility prediction [46]. The conformational B-cell epitopes were predicted by employing ElliPro (http://tools.immuneepitope.org/toolsElliPro/) [46] from the IEDB analysis resource. This analysis resource incorporates three diverse algorithms comprising protein shape approximation [47], residues protrusion index (pI) [48], and the adjacent residue clustering based on pI.

### 2.4. Potential Cytotoxic T-Lymphocyte (CTL) Epitopes Prediction

CTL epitopes were predicted by employing the NetCTL.1.2 server [49]. MHC molecules act as an antigen and utilize their surface to activate the CTLs. The NetCTL.1.2 server was employed to integrate the proteasomal C-terminal cleavage, MHC class I binding prediction, and transporter associated with antigen processing (TAP) transport efficiency. The sequences of the organism in FASTA format were submitted to the server, and afterwards, peptide lengths and human leukocyte antigen (HLA) alleles were selected and observed. Additionally, the T-cell epitope prediction and weight matrix algorithm were used for the TAP transport efficiency prediction, and artificial neural network was implemented to predict the proteasomal C-terminal cleavage and MHC class-I binding.

### 2.5. World Population Coverage Analysis

The world population coverage analysis was performed by utilizing IEDB server by utilizing the selected CTL epitopes which were searched against respective allele sets, and major world populations were covered by this analysis. The key purpose for this coverage analyses were to analyze whether the selected candidates were suitable for major populations or not. The analyses were performed against China, Iran, Japan, Korea, and some other countries which were being affected by the coronavirus in 2020 viral outbreak [50].

### 2.6. Peptide-MHC Protein Complex and Molecular Docking Studies

The predicted CTL epitope peptides of SARS-CoV-2 with antigenic residues were selected for the molecular docking analyses. The PEP-FOLD3 server [51] was employed to model the 3D structures of the selected peptides with 200 simulation runs to sample the conformations. The conformational models clustered by PEP-FOLD3 server were evaluated on the basis of sOPEP energy scores [52]. Afterwards, the peptides with higher scores were selected for molecular docking experiments with MHC class I binding molecule comprising HLA-B (PDB ID: 3VCL) through the PatchDock docking server [53]. All the docked complexes which showed the undesirable penetrations of the receptor's atoms into the ligand were rejected, and the geometric shape complementarity score was applied to classify the other complexes. Subsequently, the FireDock server [54, 55] was utilized to refine the docked complexes and also predict the score of the docking outputs.

The FireDock server supports to rectify the scoring and flexibility issues generated during the docking calculations by fast rigid-body docking tools [56]. The molecular visualization programs PyMOL [45] (Schrodinger, Inc.) and UCSF Chimera 1.11 [46] were employed to analyze and identify the hydrogen-bonding interactions of the docked complexes. The observed results suggested that the followed strategy has the capability to identify the effective epitope-based vaccines against coronavirus SARS-CoV-2 [42, 57, 58].

### 2.7. Molecular Docking Analyses

The FDA-approved library was selected for virtual screening and molecular docking analyses. The selected library has 1615 FDA-approved compounds, and all the compounds were minimized through UCSF Chimera and Chemdraw to obtain the stable configurations; all these drugs were previously derived from the ZINC database. The selected library was docked against nonstructural corona virus protein (PDB: 6LU7) involved in the replication of SARS-CoV-2 genome. The molecular docking analyses were carried out through Molecular Operating Environment (MOE) [59], AutoDock tools, and AutoDock Vina [60]. Molecular docking analyses were performed having parameters as rescoring function 1, rescoring function 2, London dG = 10, placement: triangle matcher, retain: 2, and refinement: force field = 10 for MOE. The best hits were selected based on S-score and root-mean-square deviation (RMSD) values.

The admetSAR server [61], Molinspiration [62], and Osiris explorer [63] were used to calculate the chemical and physical properties of drug-like hits. The interacting residues were analyzed and visualized through the UCSF Chimera and Ligplot tool [64].

### 2.8. MEV Construction and Molecular Docking Analyses

Replicase protein, NSP1, spikes, membrane, nucleocapsid and envelope proteins were retrieved by utilizing UniProt KB [65, 66]. HTL and CTL epitopes from the selected proteins were predicted by using the NETCTL server and ABCpred server [67]. Their physiochemical properties, antigenicity, toxicity, and immunogenicity were predicted by using the ProtParam, Vaxijen, Toxinpred, and IEDB servers, respectively [68, 69]. An adjuvant-based MEV construct was designed manually by using the selected 28 epitopes, and 3D structures were predicted by using RaptorX [70]. Structure validation was carried out by the SAVES server, and the refined structures were docked with TLR3 and TLR8 by using the HADDOCK server [71, 72].

## 3. Results

The viral pneumonia with unknown etiology had an outbreak recently in Wuhan, China [13]. Severe acute respiratory syndrome (SARS), Middle East respiratory syndrome (MERS), influenza virus, and adenovirus were not involved in the outbreak of viral pneumonia [73]. The virological.org sequenced the viral RNA genome, and World Health Organization (WHO) [74] reported the designation on 10th of January 2020. Based on genetic properties, the Coronavirinae family consists four genera including alpha-coronavirus, genus beta-coronavirus, genus gamma-coronavirus, and genus delta-coronavirus (Supplementary 1) [75].

CoVs have considered as minimal responsible pathogens causing “colds” in humans. Two extremely pathogenic CoVs named as SARS-CoV and MERS-CoV were emerged from the livestock reservoirs and caused deadly outbreaks in the 21st century. A new strain of CoV was identified named as SARS-CoV-2 in Wuhan city on December 31st, 2019. Due to the rapid changing situation, the final dimension and impact of this outbreak are currently uncertain [76]. The novel virus infects the host cells rapidly, proven through recombination of various genome practices. For this infection, no reliable mediation is currently available. The preventative measures are urgently needed due to the significant global disease burden resultant of SARS-CoV-2 [77]. A variety of tools and servers have resulted through recent advancement in immunological bioinformatics, which lessens the time and cost of traditional vaccine advancement. The development of an effective multiple-epitope vaccine remains difficult, due to problems in the selection of suitable antigen candidates and immune-dominant epitopes. Thus, it is important to predict the appropriate antigen epitopes of a targeted protein by immune-informatics approaches for designing a multiple-epitope vaccine [48]. The main target is to use immune-informatics approaches and the prediction of peptide vaccine through recognizing CTL epitopes. The discovery of novel vaccines is possible through pathogenomics analyses on a genome wide scale, though these conventional experimental methods have multiple limitations [78]. To analyze the complete spectrum of the potential antigen, immune-informatics approaches help, and furthermore, complications regarding *in vitro* expression of antigen and pathogen culturing can also be evaded. By means of computational methods, the immune research groups have reported various vaccine candidates, having promising preclinical outputs [79]. In current efforts, CTL epitopes have been identified to design the peptide vaccine against HLA-B protein [80]. The development of epitope-based vaccines targets the structural proteins of SARS-CoV-2, and CTL epitopes of the target proteins were predicted to support the host's immune response. One nonstructural protein (PDB: 6LU7) stands with the reason to use this nonstructural protein due to involvement in the replication of the virus [81–87]. The antigenicity and allergenicity of CTL epitopes were observed through Vaxijen and Allergen F.P 1.0 [88]. The population coverage estimation of predicted epitopes was calculated, and 0.5639 coverage with average hits of 4.0 for MHC class I and 0.2462 coverage with average hits of 0.91 for MHC class II (Table 1) were observed in China. The peptides were designed against eight epitopes by utilizing PEP-FOLD3. The molecular docking analyses of the selected eight peptides were performed through PatchDock and further refined through FireDock [53–55] to identify the effective binding sites.

### 3.1. Surface Accessibility Analysis for SARS-CoV-2

**Figure 2:**
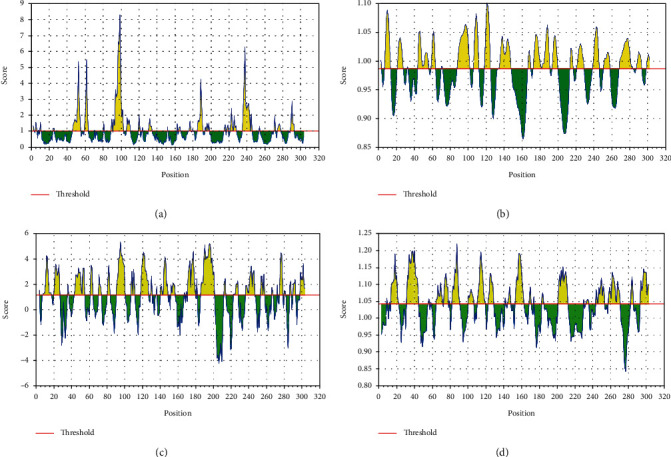
Surface accessibility, surface flexibility, Parker's hydrophilicity, and antigenicity predictions evaluated by the IEDB server for nonstructural protein (PDB: 6LU7) representing the surface probability scores of the residues (a–d), respectively. Sequence positions are represented along the *x*-axis while probability scores are represented along the *y*-axis.

A peptide with surface accessibility probability of >1.0 reflects more probable chances for a peptide to be found on the surface [43]. Numerous peptides were predicted, and the top-ranked predicted peptides of SARS-CoV-2 on the basis of surface probability (*y*-axis) and sequence position (*x*-axis) were selected for further analyses (Figure 2(a)). The maximum surface probability score of 8.254 was observed that ranges from 97 to 102 amino acids with the hexapeptide sequence of KTPKYK, while the lowest score was 0.285 from 246 to 251 residues with the hexapeptide sequence of HVDILG (Supplementary 2).

### 3.2. Surface Flexibility for Protein SARS-CoV-2

The Karplus and Schulz flexibility method was utilized to calculate and analyze the atomic vibrational motions in the protein structure designated through B-factor and temperature. The stability and organization of the structure depend upon the B-factor values. The quality of the predicted models depends upon the B-factor values as a lower B-factor value is considered an effective model while higher B-factor values lead to the less-organized and poorly ordered structures [44]. The surface flexibility outputs for SARS-CoV-2 were critically analyzed (Figure 2(b)), and it was observed that the minimum and maximum flexibility scores were 0.983 and 1.082 with the heptapeptide sequences of 129 AMRPNFT 135 and 106 IQPGQTF 112, respectively (Supplementary 2).

### 3.3. Parker Hydrophilicity Prediction for SARS-CoV-2

The hydrophilicity scale process of Parker was carried out to observe the peptides hydrophilicity based on the peptide retention times through HPLC on reversed phase column. Immunological analyses have revealed the association of antigenic sites with the hydrophilic regions [43]. Parker's hydrophilicity of SARS-CoV-2-predicted peptides in graphical form was analyzed (Figure 2(c)), where hydrophilicity is plotted along the *y*-axis and residues position is plotted along the *x*-axis.

It was observed that the Parker hydrophilicity prediction has a maximum hydrophilicity score of 5.329 which ranges from 92 to 98 with the sequence of heptapeptide 92 DTANPKT 98 while the minimum hydrophilicity score was -4.257 which ranges from 204 to 210 with the peptide sequence 204 VLAWLYA 210 (Supplementary 2).

### 3.4. Kolaskar and Tongaonkar Antigenicity Prediction for SARS-CoV-2

The antigenicity of SARS-CoV-2 was calculated through the Kolaskar and Tongaonkar method (Figure 2(d)), the maximum antigenicity values for two top-ranked peptides were observed as 1.197 for VVYCPRH and VYCPRHV at positions 35 to 41 and 36 to 42, respectively, and the minimum predicted antigenicity was 0.844 for NGMNGRT from position 274 to 280 (Supplementary 2).

### 3.5. Structure-Based Epitope Prediction for SARS-CoV-2

The correlation among the protein structure antigenicity, epitope prediction, accessibility, and flexibility within 3D structure was determined through ElliPro [89]. The significant properties including protein-antibody interactions were analyzed to differentiate the predicted epitopes. The five top-ranked conformational epitopes for SARS-CoV-2 having ≥0.6 score were observed and selected for further analyses. The pI (isoelectric point value) [89] score was observed to analyze the percentage of the atoms which extends over the molecular bulk and also liable for the antibody binding. The pI value 5.95 was observed for 6LU7. The six top-ranked conformational predicted epitopes along with residues name, length, and locations were critically analyzed (Table 2), and the score was observed between 0.51 and 0.78.

### 3.6. Molecular Docking Analyses of SARS-CoV-2 with HLA-B

The comparative molecular docking analyses were executed for 8 top-ranked selected CTL epitopes of SARS-CoV-2 out of 87 designed peptides with MHC class I HLB. The strong binding affinities have been observed for all the selected CTL epitopes having Van der Waals (VdW) energy values ranging from -23.45 to -32.62 kcal/mol, and the observed global energy was -29.63 to -50.38 kcal/mol (Table 3). The molecular docking analyses of the 8 selected CTL predicted epitopes (GTDLEGNFY, TVNVLAWLY, GSVGFNIDY, QTFSVLACY, DYDCVSFCY, TANPKTPKY, SEDMLNPNY, and LLEDEFTPF) were carried out, and effective binding affinities with HLA-B were observed.

**Figure 3:**
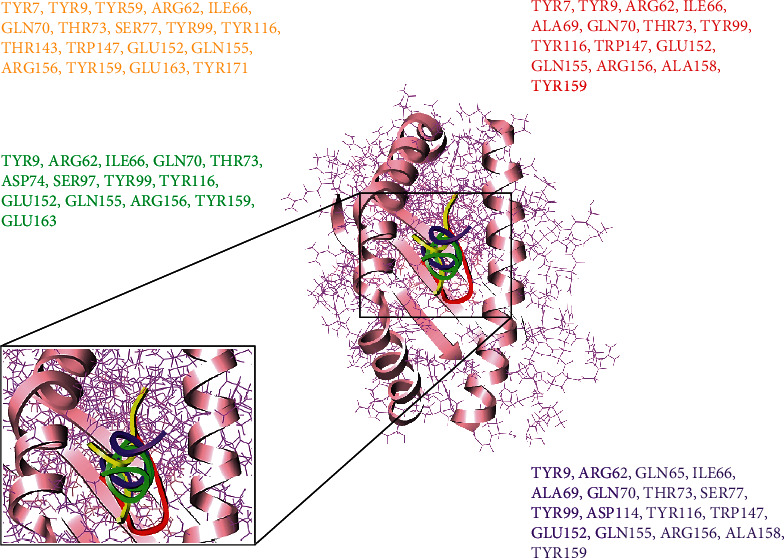
Peptide-MHC class I HLA-B binding interacting residues of four top-ranked peptides represented in different colors.

The top-ranked four docked complexes were visualized (Figure 3), and similar binding pocket has been observed in all the selected peptides. It was observed that Tyr9, Ile66, Gln70, Tyr99, Tyr116, and Arg156 residues were conserved in all the selected peptides.

### 3.7. Population Coverage Analyses

The population coverage analyses were performed with the selected MHC class I and MHC class II epitopes and also with the associated HLA alleles. It was observed that the selected MHC class I and MHC class II epitopes have the world's population of 58.49% and 34.71%, respectively. MHC class I epitopes showed highest coverage in the population of Italy (0.9019%) and China (0.5639%). The MHC class II epitopes also showed highest coverage in Philippines (0.7192%) (Supplementary 3).

### 3.8. Multiple Sequence Alignment

Multiple sequence alignment (MSA) of three coronavirus genomes were performed, and conserved binding residues were detected. It was observed that all the selected strains of coronavirus have conserved domains, reconciling with the latest outbreak strain SARS-CoV-2. Interestingly, it was observed that the reported binding domain of previously reported strain has similar region of binding with the latest outbreak of Coronavirus 2019. The binding residues of SARS-CoV-2 showed similar binding domain with the MERS and SARS (Supplementary 4).

### 3.9. Comparative Molecular Docking Analyses

**Figure 4:**
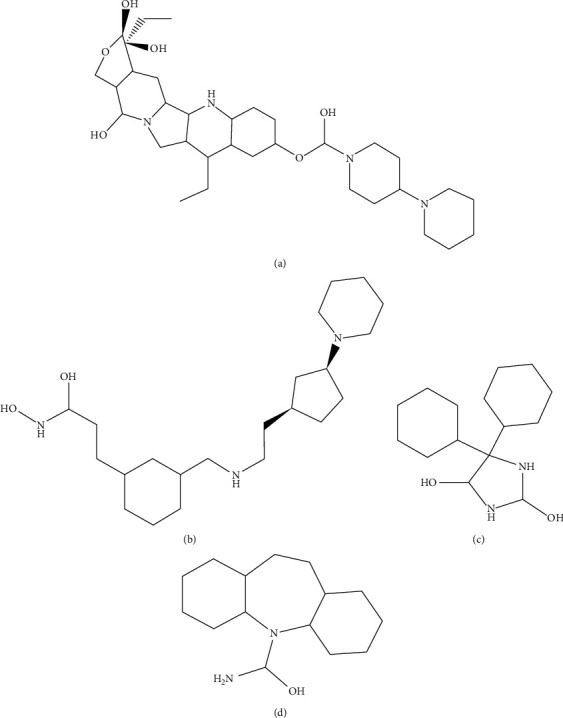
Four selected FDA-approved drugs (a) FDA-7, (b) FDA378, (c) FDA670, and (d) FDA592.

The *in silico* analyses revealed that the selected peptides have significant values against SARS-CoV-2. The comparative molecular docking analyses have been performed against the selected library of ZINC database. The molecular docking analyses showed variations in their binding energies. The FDA library (1615 compounds) [90] of ZINC database was screened through molecular docking analyses. The comparative molecular docking analyses were carried out on the selected library of 1615 compounds by using MOE, AutoDock tools, and AutoDock Vina. The blind and targeted docking was performed for the complete library (FDA library) against the selected protein. The common top-ranked compounds from blind and targeted docking were selected for further analyses. All the observed complexes of the compounds were ranked on the basis of interacting residues, highest binding affinities, drug properties, and least binding energy. The nine top-ranked docked complexes collectively from all the selected tools and docking approaches were critically visualized and analyzed. It was observed that the molecules FDA-7, FDA-378, FDA-499, and FDA-1262 (Figure 4) from the selected library were common from each selected docking tool and docking approach having least binding energies (Table 4). Almost all the docked compounds from the FDA library bound on similar binding site. The four top-ranked complexes were elucidated (Figure 4), and similar binding pocket was revealed in comparison with molecular docking analyses. The selected compounds may have the potential to inhibit the replication of SARS-CoV-2. It was elucidated that all the compounds bound at the domain II of SARS-CoV-2.

**Figure 5:**
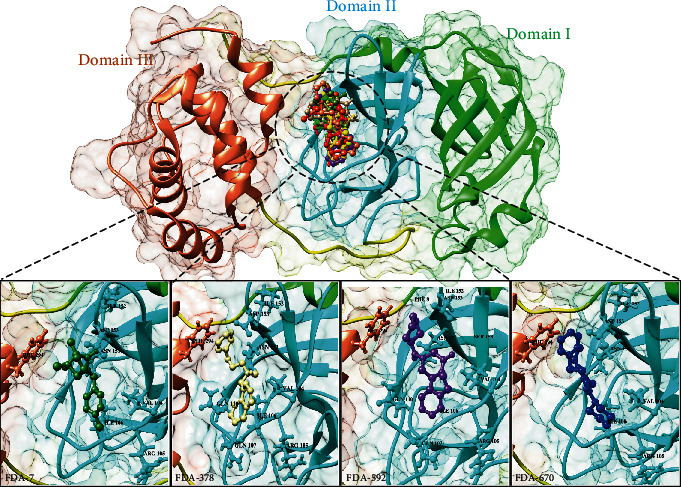
Nonstructural protein (PDB: 6LU7) has three domains, domain I from 8-99a.a (green), domain II from 100-183a.a (cyan), and domain III from 200-306a.a (brown). A conserved binding pocket present in domain II is observed while docked with FDA ligands. Top 4 ligands from FDA library have conserved interacting residues, FDA-7 (olive green), FDA-378 (skin), FDA-592 (purple), and FDA-670 (blue).

It was observed that Asp153, Phe294, Ile152, Asn151, Val104, Arg105, Gln107, Gln110, and Ile106 residues showed effective binding interactions with all the docked compounds of the FDA library. In an effort to understand the insights of the binding interactions between the docked compounds and amino acid residues of SARS-CoV-2, a plot of interactional analyses was generated by utilizing Ligplot and UCSF Chimera (Figure 5).

The FDA library has all the compounds approved by the FDA and utilized for different diseases. The FDA library's aim was to select the available compounds to inhibit the replication of SARS-CoV-2 in minimal time frame. Molinspiration, admetSAR online server, and Osiris explorer were utilized for absorption, distribution, metabolism, excretion, and toxicity (ADMET) analyses of the selected compounds (Table 4). The aqueous solubility prediction (defined water at 25°C) of the selected library revealed that the scrutinized molecules can be soluble in water. It was observed that the compounds have the ability to follow Lipinski's rule of five and also have less values of LogP involved in effective oral bioavailability. All the selected nine compounds showed similar binding site and highest binding affinity (Supplementary 5).

### 3.10. Target Protein Sequence and Structure Prediction

The amino acid sequences of SARS-CoV-2 vaccine-target proteins (replicase protein, NSp1, envelope, membrane, nucleocapsid, and spike protein) were retrieved and saved in FASTA format. The VaxiJen server was used to analyze the antigenicity of the selected proteins. Spike protein was observed as the most antigenic protein, followed by E, M, NSp1, N, and replicase proteins with antigenic values of 0.7185, 0.6502, 0.6441, 0.6131, 0.6025, and 0.5102, respectively. The 3D models of the selected proteins were predicted in order to select the suitable quality models, and the predicted structures were further refined by galaxy refine server followed by the Ramachandran plot validations. Therefore, good-quality models were selected for further analyses. There was no suitable structure predicted for spike protein because of the small number of residues.

### 3.11. HLA-B7 Allele and Epitope Interaction Analyses

To construct a subunit vaccine, the selected epitopes should be 100% conserved, overlapping, and antigenic [91, 92]. Therefore, a total of 50 conserved/antigenic epitopes from the selected proteins overlapping in all 3 categories (B-cell, T-cell, and IFN-*Γ*) were selected for further validation of their interactions with a common human allele. The 3D structures of the selected epitopes were predicted by using PEP-FOLD. The binding patterns of the selected epitopes with a common conserved allele HLA-B7 were analyzed through molecular docking, and it was found that only 28 epitopes bound deep inside in the HLA-B7 binding pocket. Each bound epitope to HLA-B7 depicts stronger than -10.00 kcal/mol docking affinity. All the 28 selected epitopes showed their binding efficiency as well as their suitability to be used in multipl-epitope-based vaccine construct (Table 5).

### 3.12. Construction of Multiepitope-Based Vaccine

**Figure 6:**
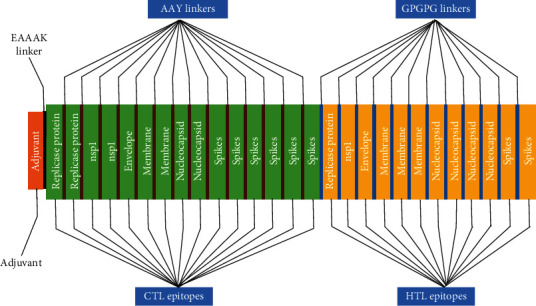
A multiepitope vaccine construct led by an adjuvant and all epitopes joined with linkers.

All 28 selected epitopes (replicase 3, NSp1 3, envelope 2, membrane 5, nucleocapsid 6, and spikes 9) were analyzed for inter-interactions and further used to develop an MEV construct. An adjuvant (45 amino acid long ß defensin) was linked with the help of EAAAK linker at the start (to the N-terminal of the MEV). The EAAAK linker reduces the interaction with other protein regions with efficient separation and increases the stability. The immunogenicity of the vaccine may increase with an adjuvant. Epitopes were merged together based on their interactional compatibility in sequential manner with AAY and GPGPG linkers, respectively. AAY and GPGPG prevent the generation of junctional epitopes, which is a major concern in the design of multiepitope vaccines. Contrarily, multiepitope vaccines facilitate the immunization and presentation of the epitopes. The final vaccine construct comprises of 479 amino acids (Figure 6).

### 3.13. Evaluation of Multiepitope Vaccine

BlastP was performed for the proteome of *Homo sapiens*, and it was observed that MEV is nonhomologous. Proteins having less than 37% identity was generally considered nonhomologous [93, 94]. However, MEV showed no similarity (higher or equal to 37%) with the proteins of human. The allergenicity, antigenicity, and toxicity of the vaccine construct were evaluated. It was observed that MEV is highly antigenic (0.6741 at 0.5% threshold), nonallergenic, and nontoxic. Furthermore, the physiochemical properties of the SARS-CoV-2 MEV construct were determined by using ProtParam. It contains 479 amino acids with 55426.35 KDa of molecular weight, indicating good antigenic nature. The isoelectric point (pI) of MEV was 9.12 showing the negative behavior. The negatively charged MEV showed the value of pI less than 7. MEV was categorized as stable as the instability index was 33.41. The aliphatic index was 82.75 showing the proportional volume of the aliphatic side chains. The protein sequence has a GRAVY value of 0.105, indicating the hydrophobic nature of the MEV. The half-life of the protein was calculated as >20 hours for yeast, 30 hours for mammalian-reticulocytes, and >10 hours for *E. coli*.

### 3.14. Structural Analyses of Multiepitope-Based Vaccine

**Figure 7:**
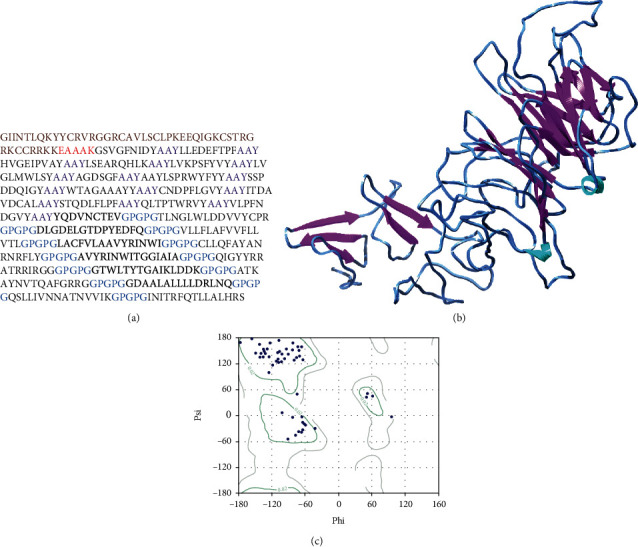
Sequence (a) elaborating the linkers (AAY, GPGPG, and EAAAK with purple, blue, and red color, respectively) Brown color adjuvant is also mentioned. MEV 3D structure is displayed (b); purple color indicates beta-sheets cyan color for loops, and the rest of blue color indicates turns in MEV. The Ramachandran plot evaluation of MEV is also elaborated (c).

The secondary structure of MEV was predicted, and from 479 amino acids, *α*-helixes were comprised of 156 amino acids representing 35.20%, 99 amino acids in *β*-strands representing 21.59%, and 215 amino acids forms the coils (42.58%) of the MEV construct. To determine the tertiary structure of the vaccine, RaptorX was used and the structure was refined by Galaxy (Figure 7). The selected structure showed that 96.3% amino acids were in allowed region, 3.7% of residues in permitted region, and 0.0% in outer region according to the Ramachandran plot analyses. Further analyses revealed that qRMSD was 0.428, poor rotamers were 0%, MolProbity was 1.889, clash score was 13.6, and *Z* score was -2.25. In addition, the refined structure showed 0 errors with PROCHECK validation. The refined structure showed 85.7143% of the overall quality factor through ERRAT. The results showed the reliability of the selected structure. The Ramachandran plot analyses of the predicted MEV structure showed that 96.3% of residues were present in favorable region.

### 3.15. Molecular Docking Analyses of Multiepitope-Based Vaccine against TLR3 and TLR8

**Figure 8:**
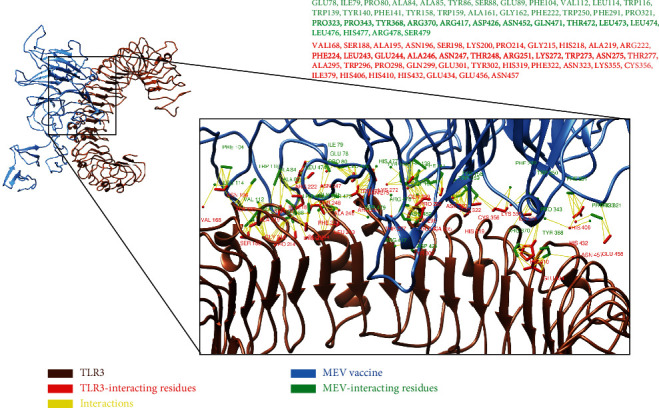
All interacting residues from MEV are shown in green color, and the rest of all red residues are TLR3-interacting residues.

An appropriate association between immune receptor molecules and the antigen molecule is essential to activate an immune responsiveness [95]. HADDOCK has been used to perform the molecular docking analyses of the MEV with human immune receptors TLR3 and TLR8. TLR3 and TLR8 can efficiently induce the immune response after virus recognition [33, 34]. The molecular docking analyses showed effective binding interactions between MEV and TLR3/TLR8. The binding scores of MEV-TLR3 and MEV-TLR8 were observed as -293.90 kcal/mol and -283.20 kcal/mol, respectively. It was observed that MEV generated 11 hydrogen bonds within the range of 3.00 Å with TLR3. MEV-interacting amino acids with hydrogen bonding to TLR3 are shown in green-colored stick representation, while similarly, TLR3 amino acids interacting through hydrogen bonding with MEV are shown in red-colored stick representation (Figure 8).

**Figure 9:**
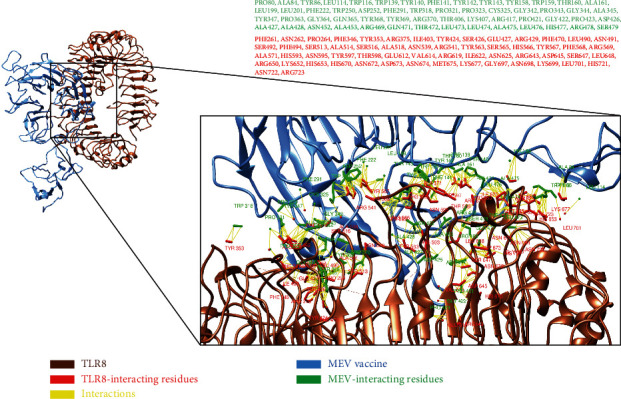
All interacting residues from MEV shown in green color and residues of TLR8 interacting residues in red color.

It was observed that MEV made 9 hydrogen bond interactions within the range of 3.00 Å with TLR8. Similar to TLR3, MEV-interacting amino acids with hydrogen bonding to TLR8 are shown in green-colored stick representation, while TLR8 amino acids interacting through hydrogen bonding with MEV are shown in red-colored stick representation (Figure 9).

## 4. Discussion

The need of dealing with coronaviruses has been increased since its recent breakout affecting millions of human lives. This SARS-CoV-2 viral outbreak became an emergency in different regions of the world [96]. As an immediate response, numerous efforts have been made to design the peptide-based vaccine against SARS-CoV-2. Peptide inhibitors are of great interest to develop vaccines [97, 98]. The peptide targets are more superior than traditional ligand-based drugs including less toxicity, fewer side-effects, and their ultrafast action. Immunoinformatics methodologies are helping researchers by reducing the workload of laboratory trials; additionally, these approaches are less time-consuming and cost-efficient than traditional approaches [99–101]. Since the last decade, there has been much progress in *in silico* drug designing [102]. Numerous biological complications are being solved by the implementation of different bioinformatics approaches [80, 102, 103].

The potential CTL epitopes have been predicted for nonstructural protein (PDB: 6LU7) of SARS-CoV-2. The molecular docking tools are applied to analyze MHC-1 and ligand-binding affinities for the selected peptides [104]. Other evidences like C-terminal cleavage affinities also validate the binding affinity of peptide-MHC-I complexes. In this study, eight peptides were reported as the potential targets with effective MHC-I protein (HLA-B) interactions. Based on global energy scores, four peptides were selected having maximum binding affinities and antigenicity, increasing the probability of the potential vaccine targets for the observed residues to be a promising target. Surface accessibility and surface flexibility, as well as hydrophobicity and antigenicity, for SARS-CoV-2 nonstructural protein were calculated and cross-verified using the IEDB server [105]. Based on an extensive literature review, it was observed that the selected peptides were not reported against SARS-CoV-2. The predicted peptides were modeled through PEP-FOLD3 server and docked to MHC-1 using PatchDock and further refined with FireDock. PyMOL and UCSF Chimera 1.11 were used to analyze the interactions of the docked complexes [46].

The *S*-value is a scoring function based upon the affinity of the ligand with the receptor [59]. The compounds having higher *S*-value with lower values of RMSD can be developed as potential inhibitors for a target protein [106]. For further evaluation, the binding energy of these selected hits were identified. The binding affinity showed the polar interaction of the hits with the binding site of receptor, and the value observed between 5 and 15 kcal/mol is considered a strong interaction among the ligands and the receptor [107, 108]. The molecular docking was also carried out using AutoDock and AutoDock Vina [109, 110].

Multiepitope vaccine construct revealed effective binding affinities against TLR3 and TLR8. The construct contains multiple epitopes from replicase, NSp1, N, E, M, and S coronavirus proteins. Various studies have been conducted by using immunoinformatics approach leading to efficient results [111–115].

## 5. Conclusion

The aim of our work was to identify the effective peptide-based inhibitors against SARS-CoV-2 nonstructural protein (PDB: 6LU7), which plays an important role in viral genome replication. Epitopes were designed, and then molecular docking was performed against MHC-I; interactional analyses of the selected docked complexes were carried out. In conclusion, four CTL epitopes (GTDLEGNFY, TVNVLAWLY, GSVGFNIDY, and QTFSVLACY) and four FDA-scrutinized compounds indicated potential targets as a peptide vaccine and potential biomolecule against deadly SARS-CoV-2, respectively. On the other hand, a multiepitope vaccine was also designed using different epitopes of coronavirus proteins joined by linkers and led by an adjuvant, which can be a possible potential MEV against coronavirus. Our findings can be a step towards the development of a peptide-based vaccine or natural compound drug target against SARS-CoV-2 which is one of the trending issues nowadays due to the exponentially increasing death rate all over the world.

## Figures and Tables

**Table 1:** Predicted CTL epitopes from the SARS-CoV-2 and predicted amino acid residues (in bold) having antigenic sites.

Residue number	Peptide sequence	Predicted MHC binding affinity	Rescale binding affinity	C-terminal cleavage affinity	TAP transport efficiency	Prediction score
174	GTDLEGNFY	0.793	3.3669	0.6229	2.702	3.5954
201	TVNVLAWLY	0.6255	2.6559	0.8852	2.957	2.9365
146	GSVGFNIDY	0.3112	1.3211	0.9565	2.857	1.6075
110	QTFSVLACY	0.2625	1.1146	0.9725	2.998	1.4104
153	DYDCVSFCY	0.2097	0.8905	0.9722	2.706	1.1717
93	TANPKTPKY	0.1676	0.7118	0.9755	2.723	0.9942
46	SEDMLNPNY	0.1528	0.6489	0.8406	2.676	0.9088
286	LLEDEFTPF	0.1132	0.4807	0.9503	2.568	0.7517

**Table 2:** Top-ranked selected discontinuous epitopes, interacting residues, and scores.

Predicted discontinuous epitopes			
Sr. No.	Residues	Number of residues	Score
1	A:R40, A:C44, A:T45, A:S46, A:E47, A:D48, A:M49, A:L50, A:N51, A:P52, A:N53, A:Y54, A:D56, A:L57, A:I59, A:R60, A:V186, A:D187, A:R188, A:Q189, A:T190	21	0.784
2	A:Q244, A:D245, A:V247, A:D248	4	0.725
3	A:S1, A:G2, A:F3, A:T198, A:V212, A:I213, A:N214, A:G215, A:D216, A:R217, A:W218, A:F219, A:L220, A:N221, A:R222, A:F223, A:T224, A:T225, A:T226, A:L227, A:N228, A:D229, A:F230, A:N231, A:L232, A:V233, A:A234, A:M235, A:K236, A:Y237, A:N238, A:Y239, A:E240, A:P241, A:L242, A:T243, A:G251, A:P252, A:S254, A:A255, A:Q256, A:T257, A:G258, A:I259, A:A260, A:L262, A:D263, A:A266, A:S267, A:K269, A:E270, A:L271, A:L272, A:Q273, A:N274, A:G275, A:M276, A:N277, A:G278, A:R279, A:T280, A:I281, A:L282, A:G283, A:S284, A:A285, A:L286, A:S301, A:G302, A:V303, A:T304, A:F305, A:Q306	73	0.712
4	A:G11, A:K12, A:G15, A:C16, A:T21, A:C22, A:G23, A:T24, A:T26, A:D33, A:D34, A:E55, A:L58, A:K61, A:S62, A:N63, A:H64, A:N65, A:L67, A:Q69, A:A70, A:G71, A:N72, A:V73, A:Q74, A:L75, A:R76, A:V77, A:I78, A:G79, A:H80, A:S81, A:K90, A:V91, A:D92, A:T93, A:A94, A:N95, A:P96, A:K97, A:T98, A:P99, A:K100, A:N119, A:G120, A:D155, A:C156	47	0.707
5	A:G183, A:P184, A:F185, A:A191, A:Q192, A:A193, A:A194	7	0.552
6	A:L167, A:P168, A:T169, A:V171	4	0.521

**Table 3:** Summary of designed peptides against SARS-CoV-2 peptides-MHC class I HLA-B interactions.

Peptide	Global energy (kcal/Mol)	Attractive VdW energy (kcal/Mol)	H-Bond energy (kcal/Mol)	Peptidase-MHC pair	Bond distance (Å)	Conserved residues
GTDLEGNFY	-43.24	-28.87	0.22	PHE8 O-ARG156 A THR2 O-ILE66 CD1 THR2 N-ARG62 NH2 ASN7 OD1-TYR99 OH TYR9 CZ-TRP147 CE2 SER4 CB-ILE66 CD1	2.020 2.474 2.575 1.319 2.194 2.315	TYR9 ARG62 ILE66 THR73 TYR99 GLU152
TVNVLAWLY	-50.38	-32.3	-3.03	LEU8 C-TYR99 OH LEU8 O-TYR99 OH ALA6 O-ILE66 HG22 TRP7 CG-GLN70 CD TYR9 O1-IL66 CG2 SER4 CB-ILE66 CD1	2.573 1.960 1.768 2.688 2.497 1.596	TYR9 ARG62 ILE66 THR73 TYR99 GLU152
GSVGFNIDY	-42.49	-27.33	-1.15	PHE5 O-THR73 OG1 PHE5 O-THR73 CB SER2 O-ILE66 CG2 ASP8 OD2-TYR99 CD1 ASN6 N-THR73 CG2 GLY4 CA-GLN70 OE1	0.425 1.321 2.103 2.144 2.559 2.698	TYR9 ARG62 ILE66 THR73 TYR99 GLU152
QTFSVLACY	-40.01	-23.86	-1.887	TYR9 C-TYR116 OH LEU6 O-ARG156 HD3 SER4 CB-ILE66 CD1 CYS8 CB-TYR99 OH GLN1 OE1-ILE66 N CYS8 O-TYR99 CD1	1.475 1.634 2.744 2.682 2.011 2.493	TYR9 ARG62 ILE66 THR73 TYR99 GLU152
DYDCVSFCY	-40.48	-26.48	-1.2	CYS8 CB-GLN70 OE1 PHE7 CZ-ARG62 NH1 CYS8 SG-GLN70 OE1 ASP1 CG-GLU154 O GLN1 OE1-ILE66 N CYS8 O-TYR99 CD1	0.952 1.159 1.542 2.571 2.253 1.693	TYR9 ARG62 ILE66 THR73 TYR99 GLU152
TANPKTPKY	-32.96	-23.45	-1.65	TYR9 C-THR73 OG1 LYS8 O-THR73 HG21 PRO7 CD-TRP147 CZ2 LYS5 CE-TYR99 OH THR6 CA-TYR116 HH	1.336 0.712 2.509 2.317 2.027 2.693	TYR9 ARG62 ILE66 THR73 TYR99 GLU152
SEDMLNPNY	-29.63	-26.6	-0.72	SER1 CB-THR73 OG1 LEU5 CD2-GLN70 OE1 MET4 CE-ILE66 CA TYR9 O1-TYR159 HB2	0.732 1.252 2.377 1.283 1.986 2.563	TYR9 ARG62 ILE66 THR73 TYR99 GLU152
LLEDEFTPF	-35.13	-32.62	-3.99	PRO8 CB-THR73 OG1 LEU1 N-ARG156 CD GLU5 O-ILE66 HG22 SER1 CB-THR73 OG1 LEU5 CD2-GLN70 OE1 MET4 CE-ILE66 CA	1.679 1.813 1.750 1.569 2.576 2.201	TYR9 ARG62 ILE66 THR73 TYR99 GLU152

**Table 4:** Four FDA ligands selected by molecular docking studies and their properties evaluated by MOE, AutoDock, AutoDock Vina, and admetSAR.

Ligands	Binding energy AD (kcal/Mol)	*S*-score MOE (kcal/Mol)	RMSD value	Molecular weight (g/Mol)	A-logP value	H-Bond acceptor	H-Bond donor	Rotatable bond	Water solubility (logS)	Acute oral toxicity (kg/Mol)	Interacting residues	Lipinski's rule of five violation
FDA-7	-7.0	-9.9153	1.7917	606.85	1.51	10	5	6	-3.015	4.709	VAL104 ARG105 ILE106 ASN151 PHE294	03
FDA-378	-7.9	-9.4894	1.9330	367.58	3.02	5	5	9	-2.598	2.964	PHE8 VAL104 ARG105 GLN107 GLN110 ASN151 ILE152 ASP153 SER158 PHE294	00
FDA-670	-7.8	-9.3083	1.8382	268.40	1.67	4	4	2	-2.993	3.251	VAL104 ARG105 GLN107 GLN110 ASN151 ASP153 PHE294	00
FDA-592	-7.6	-8.6105	1.8580	265.44	2.57	3	3	0	-2.579	3.738	VAL104 ILE106 GLN110 ASN151 ASP153 SER158 PHE294	00

**Table 5:** Selected epitopes for MEV along with their antigenicity, binding affinities, and other properties.

Sr. No.	Protein	Epitopes	Antigenicity	Binding score (kcal/Mol) with HLA-B7	Predicted MHC binding affinity	Rescale binding affinity	C-terminal cleavage affinity	TAP transport efficiency	Position
MHC class I									
1	Nsp1	HVGEIPVAY	0.81	-11.76	1.193	4.366	0.229	1.702	37-45
2	Nsp1	LSEARQHLK	0.16	-11.55	0.325	3.659	0.852	2.957	60-68
3	Replicase	GSVGFNIDY	1.52	-14.25	1.212	0.311	0.955	0.857	12-21
4	Replicase	LLEDEFTPF	2.37	-10.22	1.651	2.146	0.972	3.998	31-39
5	Envelope	LVKPSFYVY	0.63	-10.36	1.297	3.905	0.942	2.706	9-17
6	Membrane	LVGLMWLSY	0.54	-10.87	0.176	2.718	0.755	0.723	54-62
7	Membrane	AGDSGFAAY	0.52	-15.00	0.158	2.649	0.806	1.676	93-101
8	Nucleocapsid	LSPRWYFYY	0.87	-14.78	0.113	2.480	0.973	2.518	99-107
9	Nucleocapsid	SSPDDQIGY	0.65	-13.57	0.693	0.369	0.621	2.602	154-162
10	Spikes	WTAGAAAYY	0.35	-12.22	0.625	1.659	0.892	2.937	27-35
11	Spikes	CNDPFLGVY	1.32	-12.63	0.812	6.211	0.365	2.857	59-67
12	Spikes	ITDAVDCAL	1.52	-15.21	0.713	3.369	0.629	2.700	71-79
13	Spikes	STQDLFLPF	0.57	-11.36	0.631	0.651	0.880	2.857	88-96
14	Spikes	QLTPTWRVY	2.0	-10.28	0.302	2.611	0.915	2.352	112-120
15	Spikes	VLPFNDGVY	1.70	-14.27	0.005	3.106	0.025	2.908	137-145
16	Spikes	YQDVNCTEV	0.08	-14.26	0.117	2.835	0.932	2.716	199-207
MHC class II									
17	Nsp1	DLGDELGTDPYEDFQ	0.12	-11.32	0.693	2.366	0.663	2.976	69-83
18	Replicase	TLNGLWLDDVVYCPR	0.77	-12.88	0.723	0.659	0.872	2.126	101-115
19	Envelope	VLLFLAFVVFLLVTL	2.52	-11.01	1.556	3.311	0.365	2.357	99-113
20	Membrane	LACFVLAAVYRINWI	1.37	-13.73	1.327	2.146	0.985	2.256	127-141
21	Membrane	CLLQFAYANRNRFLY	0.33	-14.58	0.786	2.805	0.900	2.799	196-210
22	Membrane	AVYRINWITGGIAIA	0.55	-10.27	0.456	1.718	0.002	2.159	222-236
23	Nucleocapsid	QIGYYRRATRRIRGG	0.83	-10.66	0.551	4.648	0.116	2.015	13-27
24	Nucleocapsid	GTWLTYTGAIKLDDK	1.54	-13.22	1.007	3.487	0.963	2.367	47-61
25	Nucleocapsid	ATKAYNVTQAFGRRG	1.12	-13.37	1.697	0.369	0.129	2.449	68-82
26	Nucleocapsid	GDAALALLLLDRLNQ	2.54	-15.24	0.273	0.559	0.652	2.441	171-185
27	Spikes	QSLLIVNNATNVVIK	1.02	-10.25	0.123	2.311	0.756	2.221	9-23
28	Spikes	INITRFQTLLALHRS	2.38	-12.16	0.357	3.116	0.925	2.118	166-180

## Abbreviations

CPE: Cytopathic effects
SARS-CoV: Severe acute respiratory syndrome coronavirus
MERS-CoV: Middle East respiratory syndrome coronavirus
RBD: Receptor-binding domain
MHC: Major histocompatibility complex
HLA: Human leukocyte antigen
MOE: Molecular Operating Environment
CTL: Cytotoxic T-lymphocyte
pI: Isoelectric point
ADMET: Absorption distribution metabolism elimination toxicity.

## Supplementary Materials

Supplementary 1 Effect of SARS (severe acute respiratory syndrome) in 2002.

Supplementary 2 CTL epitopes and physiochemical properties of epitopes.

Supplementary 3 Physiochemical properties and population coverage analysis.

Supplementary 4 Multiple sequence alignment.

Supplementary 5 FDA ligands selected by molecular docking studies and their properties evaluated by MOE, AutoDock, AutoDock Vina, and admetSAR.
