# Supplementary material for: Immunoinformatics and Molecular Docking Studies Predicted Potential Multiepitope-Based Peptide Vaccine and Novel Compounds against Novel SARS-CoV-2 through Virtual Screening
Source: Biomed Res Int. 2021 Feb 26;2021:1596834. doi: 10.1155/2021/1596834 (PMC7910514; doi:10.1155/2021/1596834)
Supplement: Supplementary 1 — Effect of SARS (severe acute respiratory syndrome) in 2002. [file 1596834.f1.docx]

**Table 1: Effect of SARS (severe acute respiratory syndrome**) **in 2002**

| **Effect of SARS (severe acute respiratory syndrome**) **in 2002** | | | | | | |
| --- | --- | --- | --- | --- | --- | --- |
| No. of countries affected | | No. of deaths | | Economic loss | | No. of months |
| 37 | | 774 | | US$40 billion | | 6 |
| **Effect of n-CoV(novel coronavirus)in 2019-20 (31 December 2019 and as of 9 February 2020)** | | | | | | |
| No. of countries affected | | | No. of deaths | | No. of confirmed cases | |
| 28 | | | 813 | | 37,564 | |
| Confirmed cases of n-CoV (novel coronavirus) in 2019-20 worldwide | | | | | | |
| **Asia** | China , Hong Kong (Special Administrative Region), Macao (Special Administrative Region), Singapore, Thailand, Japan, Republic of Korea, Taiwan, Malaysia, Vietnam, United Arab Emirates, India, Philippines, Cambodia, Nepal and Sri Lanka | | | | | |
| **America** | The United States and Canada | | | | | |
| **Europe** | Germany, France, United Kingdom, Italy, Russia, Belgium, Finland, Spain and Sweden. | | | | | |
| **Oceania** | Australia | | | | | |
| **Other** | Cases on an international conveyance  Japan | | | | | |

| **COUNTRY** | **REPORTED CASES** |
| --- | --- |
| China (PRC) (37 206) | 37206 |
| Hong Kong (Special Administrative Region) | 26 |
| Macao (Special Administrative Region) | 10 |
| Singapore | 40 |
| Thailand | 32 |
| Japan | 26 |
| Republic of Korea | 25 |
| Taiwan | 18 |
| Malaysia | 16 |
| Vietnam | 14 |
| United Arab Emirates | 7 |
| India | 3 |
| Philippines | 3 |
| Cambodia | 1 |
| Nepal | 1 |
| Sri Lanka | 1 |
| United States | 12 |
| Canada | 7 |
| Germany | 14 |
| France | 11 |
| United Kingdom | 3 |
| Italy | 3 |
| Russia | 2 |
| Belgium | 1 |
| Finland | 1 |
| Spain | 1 |
| Sweden | 1 |
| Australia | 15 |

**Table 2: Confirmed cases of n-CoV acute respiratory disease reported by cities and regions in China 2019-20**

| **Effect of n-CoV (novel coronavirus) in china 2019-20** | | | |
| --- | --- | --- | --- |
| Confirmed cases | | Deaths | |
| 37,206 | | 811 | |
| **Confirmed cases and Deaths by provinces, regions and cities in China** | | | |
| **province/Regions/cities** | **Confirmed cases** | | **Deaths** |
| Hubei | 19 665 | | 780 |
| Heilongjiang | 227 | | 6 |
| Henan | 851 | | 6 |
| Beijing | 274 | | 2 |
| Chongqing | 389 | | 2 |
| Hainan | 100 | | 2 |
| Hebei | 157 | | 2 |
| Anhui | 591 | | 1 |
| Gansu | 62 | | 2 |
| Guangdong | 944 | | 1 |
| Guizhou | 69 | | 1 |
| Hunan | 711 | | 1 |
| Jilin | 59 | | 1 |
| Shandong | 343 | | 1 |
| Shanghai | 254 | | 1 |
| Sichuan | 321 | | 1 |
| Tianjin | 70 | | 1 |
