# Supplementary material for: Immunoinformatics and Molecular Docking Studies Predicted Potential Multiepitope-Based Peptide Vaccine and Novel Compounds against Novel SARS-CoV-2 through Virtual Screening
Source: Biomed Res Int. 2021 Feb 26;2021:1596834. doi: 10.1155/2021/1596834 (PMC7910514; doi:10.1155/2021/1596834)
Supplement: Supplementary 2 — CTL epitopes and physiochemical properties of epitopes. [file 1596834.f2.pdf]

Residue Number	Peptide Sequence	Predicted MHC Binding Affinity
174	GTDLEGNFY	0.793
201	TVNVLAWLY	0.6255
146	GSVGFNIDY	0.3112
110	QTFSVLACY	0.2625
153	DYDCVSFCY	0.2097
93	TANPKTPKY	0.1676
46	SEDMLNPNY	0.1528
286	LLEDEFTPF	0.1132

predicted by NETCTL 1.2 server
threshhold 0.75000

NetCTL-1.2 predictions using MHC supertype A1. Threshold 0.750000

174 ID 6lu7_A pep GTDLEGNFY aff 0.7930 aff_rescale 3.3669 cle 0.6229 tap 2
201 ID 6lu7_A pep TVNVLAWLY aff 0.6255 aff_rescale 2.6559 cle 0.8852 tap
146 ID 6lu7_A pep GSVGFNIDY aff 0.3112 aff_rescale 1.3211 cle 0.9565 tap 2.
110 ID 6lu7_A pep QTFSVLACY aff 0.2625 aff_rescale 1.1146 cle 0.9725 tap 2
153 ID 6lu7_A pep DYDCVSFCY aff 0.2097 aff_rescale 0.8905 cle 0.9722 tap [illegible]
93 ID 6lu7_A pep TANPKTPKY aff 0.1676 aff_rescale 0.7118 cle 0.9755 tap 2.
46 ID 6lu7_A pep SEDMLNPNY aff 0.1528 aff_rescale 0.6489 cle 0.8406 tap 2
286 ID 6lu7_A pep LLEDEFTPF aff 0.1132 aff_rescale 0.4807 cle 0.9503 tap 2.[illegible]
261 ID 6lu7_A pep VLDMCASLK aff 0.1397 aff_rescale 0.5933 cle 0.7881 tap [illegible]
231 ID 6lu7_A pep NLVAMKYNY aff 0.1073 aff_rescale 0.4555 cle 0.8757 tap
118 ID 6lu7_A pep YNGSPSGVY aff 0.0999 aff_rescale 0.4243 cle 0.9564 tap [illegible]
256 ID 6lu7_A pep QTGIAVLDM aff 0.1269 aff_rescale 0.5388 cle 0.9157 tap [illegible]
23 ID 6lu7_A pep GTTTLNGLW aff 0.1311 aff_rescale 0.5566 cle 0.4256 tap 0
253 ID 6lu7_A pep LSAQTGIAV aff 0.1426 aff_rescale 0.6054 cle 0.0941 tap 0.
24 ID 6lu7_A pep TTTLNGLWL aff 0.1293 aff_rescale 0.5489 cle 0.2161 tap 0.
225 ID 6lu7_A pep TTLNDFNLV aff 0.1106 aff_rescale 0.4694 cle 0.9195 tap 0
81 ID 6lu7_A pep SMQNCVLKL aff 0.0952 aff_rescale 0.4042 cle 0.9581 tap 1
157 ID 6lu7_A pep VSFCYMHHM aff 0.0996 aff_rescale 0.4230 cle 0.9507 tap
195 ID 6lu7_A pep GTDTTITVN aff 0.1527 aff_rescale 0.6485 cle 0.0525 tap -1.
229 ID 6lu7_A pep DFNLVAMKY aff 0.0669 aff_rescale 0.2841 cle 0.9722 tap
242 ID 6lu7_A pep LTQDHVDIL aff 0.1109 aff_rescale 0.4711 cle 0.3794 tap 0.
219 ID 6lu7_A pep FLNRFTTTL aff 0.0917 aff_rescale 0.3893 cle 0.9334 tap 0.
29 ID 6lu7_A pep GLWLDDVVY aff 0.0620 aff_rescale 0.2632 cle 0.9766 tap [illegible]
185 ID 6lu7_A pep FVDRQTAQA aff 0.1082 aff_rescale 0.4593 cle 0.7828 tap -[illegible]
151 ID 6lu7_A pep NIDYDCVSF aff 0.0733 aff_rescale 0.3111 cle 0.6732 tap 2.
279 ID 6lu7_A pep RTILGSALL aff 0.0762 aff_rescale 0.3234 cle 0.9504 tap 1.[illegible]
283 ID 6lu7_A pep GSALLEDEF aff 0.0921 aff_rescale 0.3911 cle 0.0575 tap 2.
297 ID 6lu7_A pep VRQCSGVTF aff 0.0554 aff_rescale 0.2353 cle 0.9711 tap 2
80 ID 6lu7_A pep HSMQNCVLK aff 0.0917 aff_rescale 0.3893 cle 0.6203 tap [illegible]
19 ID 6lu7_A pep QVTCGTTTL aff 0.0704 aff_rescale 0.2990 cle 0.9775 tap 1.

254 ID 6lu7_A pep SAQTGIAVL aff 0.0701 aff_rescale 0.2975 cle 0.9645 tap 1.
104 ID 6lu7_A pep VRIQPGQTF aff 0.0531 aff_rescale 0.2253 cle 0.8613 tap 2.
197 ID 6lu7_A pep DTTITVNVL aff 0.0792 aff_rescale 0.3362 cle 0.8423 tap 0.
49 ID 6lu7_A pep MLNPNYEDL aff 0.0737 aff_rescale 0.3130 cle 0.9214 tap 0
5 ID 6lu7_A pep KMAFPSGKV aff 0.0729 aff_rescale 0.3094 cle 0.9651 tap 0.
69 ID 6lu7_A pep QAGNVQLRV aff 0.0915 aff_rescale 0.3886 cle 0.5614 tap 0
223 ID 6lu7_A pep FTTTLNDFN aff 0.1278 aff_rescale 0.5426 cle 0.0225 tap -1
60 ID 6lu7_A pep RKSNHNFLV aff 0.0798 aff_rescale 0.3386 cle 0.7271 tap 0.
67 ID 6lu7_A pep LVQAGNVQL aff 0.0676 aff_rescale 0.2870 cle 0.9057 tap 0
142 ID 6lu7_A pep NGSCGSVGF aff 0.0518 aff_rescale 0.2201 cle 0.8785 tap 2
209 ID 6lu7_A pep YAAVINGDR aff 0.0880 aff_rescale 0.3735 cle 0.0818 tap 1
222 ID 6lu7_A pep RFTTTLNDF aff 0.0538 aff_rescale 0.2283 cle 0.5532 tap 3.
44 ID 6lu7_A pep CTSEDMLNP aff 0.1073 aff_rescale 0.4555 cle 0.0243 tap 0.
226 ID 6lu7_A pep TLNDFNLVA aff 0.0921 aff_rescale 0.3910 cle 0.6700 tap -0
164 ID 6lu7_A pep HMELPTGVH aff 0.0871 aff_rescale 0.3697 cle 0.8244 tap -[illegible]
224 ID 6lu7_A pep TTTLNDFNL aff 0.0908 aff_rescale 0.3855 cle 0.2364 tap 0.
260 ID 6lu7_A pep AVLDMCASL aff 0.0588 aff_rescale 0.2496 cle 0.9666 tap [illegible]
200 ID 6lu7_A pep ITVNVLAWL aff 0.0672 aff_rescale 0.2851 cle 0.7940 tap 1
128 ID 6lu7_A pep CAMRPNFTI aff 0.0687 aff_rescale 0.2918 cle 0.9030 tap 0.
215 ID 6lu7_A pep GDRWFLNRF aff 0.0490 aff_rescale 0.2080 cle 0.8800 tap [illegible]
177 ID 6lu7_A pep LEGNFYGPF aff 0.0617 aff_rescale 0.2619 cle 0.5531 tap 2.
95 ID 6lu7_A pep NPKTPKYKF aff 0.0434 aff_rescale 0.1845 cle 0.9601 tap 2.
180 ID 6lu7_A pep NFYGPFVDR aff 0.0496 aff_rescale 0.2105 cle 0.9661 tap 1
97 ID 6lu7_A pep KTPKYKFVR aff 0.0676 aff_rescale 0.2868 cle 0.5741 tap 1.
58 ID 6lu7_A pep LIRKSNHNF aff 0.0641 aff_rescale 0.2724 cle 0.2109 tap 2.7
129 ID 6lu7_A pep AMRPNFTIK aff 0.0602 aff_rescale 0.2557 cle 0.9625 tap 0
140 ID 6lu7_A pep FLNGSCGSV aff 0.0719 aff_rescale 0.3052 cle 0.7902 tap 0
106 ID 6lu7_A pep IQPGQTFSV aff 0.0609 aff_rescale 0.2586 cle 0.9756 tap 0.5
126 ID 6lu7_A pep YQCAMRPNF aff 0.0684 aff_rescale 0.2903 cle 0.0536 tap [illegible]
12 ID 6lu7_A pep KVEGCMVQV aff 0.0775 aff_rescale 0.3290 cle 0.5447 tap [illegible]
239 ID 6lu7_A pep YEPLTQDHV aff 0.0695 aff_rescale 0.2953 cle 0.7922 tap 0
211 ID 6lu7_A pep AVINGDRWF aff 0.0593 aff_rescale 0.2518 cle 0.1557 tap 3
205 ID 6lu7_A pep LAWLYAAVI aff 0.0603 aff_rescale 0.2559 cle 0.8650 tap 0
194 ID 6lu7_A pep AGTDTTITV aff 0.0643 aff_rescale 0.2730 cle 0.8827 tap 0.
204 ID 6lu7_A pep VLAWLYAAV aff 0.0794 aff_rescale 0.3369 cle 0.3587 tap
234 ID 6lu7_A pep AMKYNYEPL aff 0.0542 aff_rescale 0.2302 cle 0.8302 tap
132 ID 6lu7_A pep PNFTIKGSF aff 0.0483 aff_rescale 0.2051 cle 0.6862 tap 2.1
192 ID 6lu7_A pep QAAGTDTTI aff 0.0771 aff_rescale 0.3272 cle 0.3248 tap 0.
290 ID 6lu7_A pep EFTPFDVVR aff 0.0479 aff_rescale 0.2035 cle 0.9544 tap 1.
59 ID 6lu7_A pep IRKSNHNFL aff 0.0493 aff_rescale 0.2093 cle 0.9245 tap 1.2
92 ID 6lu7_A pep DTANPKTPK aff 0.0689 aff_rescale 0.2925 cle 0.6897 tap 0.
35 ID 6lu7_A pep VVYCPRHVI aff 0.0524 aff_rescale 0.2226 cle 0.9571 tap 0.
198 ID 6lu7_A pep TTITVNVLA aff 0.1007 aff_rescale 0.4275 cle 0.0571 tap -0.
274 ID 6lu7_A pep NGMNGRTIL aff 0.0556 aff_rescale 0.2359 cle 0.8713 tap 0

278 ID 6lu7_A pep GRTILGSAL aff 0.0483 aff_rescale 0.2050 cle 0.9609 tap 1.
74 ID 6lu7_A pep QLRVIGHSM aff 0.0581 aff_rescale 0.2467 cle 0.8998 tap 0.
212 ID 6lu7_A pep VINGDRWFL aff 0.0656 aff_rescale 0.2784 cle 0.4296 tap 1
163 ID 6lu7_A pep HHMELPTGV aff 0.0588 aff_rescale 0.2498 cle 0.8911 tap
107 ID 6lu7_A pep QPGQTFSVL aff 0.0536 aff_rescale 0.2275 cle 0.9780 tap 0
31 ID 6lu7_A pep WLDDVVYCP aff 0.0903 aff_rescale 0.3832 cle 0.1133 tap -
159 ID 6lu7_A pep FCYMHHMEL aff 0.0648 aff_rescale 0.2752 cle 0.3944 tap
245 ID 6lu7_A pep DHVDILGPL aff 0.0491 aff_rescale 0.2084 cle 0.9561 tap 0.
199 ID 6lu7_A pep TITVNVLAW aff 0.0647 aff_rescale 0.2748 cle 0.4084 tap 0
133 ID 6lu7_A pep NFTIKGSFL aff 0.0473 aff_rescale 0.2007 cle 0.8908 tap 0.
263 ID 6lu7_A pep DMCASLKEL aff 0.0637 aff_rescale 0.2706 cle 0.4633 tap
42 ID 6lu7_A pep VICTSEDML aff 0.0696 aff_rescale 0.2953 cle 0.1856 tap 1.
79 ID 6lu7_A pep GHSMQNCVL aff 0.0483 aff_rescale 0.2051 cle 0.8993 tap
41 ID 6lu7_A pep HVICTSEDM aff 0.0688 aff_rescale 0.2920 cle 0.3478 tap 0.
271 ID 6lu7_A pep LLQNGMNGR aff 0.0630 aff_rescale 0.2676 cle 0.2531 tap
264 ID 6lu7_A pep MCASLKELL aff 0.0712 aff_rescale 0.3023 cle 0.1443 tap 0
214 ID 6lu7_A pep NGDRWFLNR aff 0.0653 aff_rescale 0.2772 cle 0.2389 tap
53 ID 6lu7_A pep NYEDLLIRK aff 0.0555 aff_rescale 0.2355 cle 0.7029 tap 0.
68 ID 6lu7_A pep VQAGNVQLR aff 0.0605 aff_rescale 0.2568 cle 0.1444 tap 1
295 ID 6lu7_A pep DVVRQCSGV aff 0.0587 aff_rescale 0.2491 cle 0.7132 tap
50 ID 6lu7_A pep LNPNYEDLL aff 0.0608 aff_rescale 0.2581 cle 0.4079 tap 0.
241 ID 6lu7_A pep PLTQDHVDI aff 0.0528 aff_rescale 0.2242 cle 0.9424 tap -0
98 ID 6lu7_A pep TPKYKFVRI aff 0.0469 aff_rescale 0.1993 cle 0.9573 tap 0.
289 ID 6lu7_A pep DEFTPFDVV aff 0.0532 aff_rescale 0.2258 cle 0.9453 tap -0
251 ID 6lu7_A pep GPLSAQTGI aff 0.0522 aff_rescale 0.2218 cle 0.8450 tap 0.
196 ID 6lu7_A pep TDTTITVNV aff 0.0584 aff_rescale 0.2481 cle 0.7282 tap -0.
246 ID 6lu7_A pep HVDILGPLS aff 0.1113 aff_rescale 0.4726 cle 0.0349 tap -2.
210 ID 6lu7_A pep AAVINGDRW aff 0.0539 aff_rescale 0.2287 cle 0.4492 tap
114 ID 6lu7_A pep VLACYNGSP aff 0.0783 aff_rescale 0.3323 cle 0.0333 tap 0
169 ID 6lu7_A pep TGVHAGTDL aff 0.0506 aff_rescale 0.2147 cle 0.6985 tap
165 ID 6lu7_A pep MELPTGVHA aff 0.0570 aff_rescale 0.2421 cle 0.9187 tap -
130 ID 6lu7_A pep MRPNFTIKG aff 0.0668 aff_rescale 0.2837 cle 0.7644 tap -1
173 ID 6lu7_A pep AGTDLEGNF aff 0.0482 aff_rescale 0.2048 cle 0.1675 tap 2
156 ID 6lu7_A pep CVSFCYMHH aff 0.0842 aff_rescale 0.3577 cle 0.1177 tap -
117 ID 6lu7_A pep CYNGSPSGV aff 0.0510 aff_rescale 0.2167 cle 0.6599 tap 0
228 ID 6lu7_A pep NDFNLVAMK aff 0.0550 aff_rescale 0.2333 cle 0.6255 tap
233 ID 6lu7_A pep VAMKYNYEP aff 0.0750 aff_rescale 0.3185 cle 0.0416 tap
227 ID 6lu7_A pep LNDFNLVAM aff 0.0594 aff_rescale 0.2523 cle 0.5904 tap -
61 ID 6lu7_A pep KSNHNFLVQ aff 0.0765 aff_rescale 0.3246 cle 0.0573 tap 0
83 ID 6lu7_A pep QNCVLKLKV aff 0.0627 aff_rescale 0.2664 cle 0.3735 tap 0
268 ID 6lu7_A pep LKELLQNGM aff 0.0548 aff_rescale 0.2326 cle 0.5735 tap
265 ID 6lu7_A pep CASLKELLQ aff 0.0777 aff_rescale 0.3301 cle 0.0293 tap -0
65 ID 6lu7_A pep NFLVQAGNV aff 0.0555 aff_rescale 0.2358 cle 0.4617 tap 0
90 ID 6lu7_A pep KVDTANPKT aff 0.0834 aff_rescale 0.3542 cle 0.0364 tap -0

52 ID 6lu7_A pep PNYEDLLIR aff 0.0479 aff_rescale 0.2035 cle 0.4507 tap 1.1
22 ID 6lu7_A pep CGTTTLNGL aff 0.0548 aff_rescale 0.2327 cle 0.4402 tap 0.
161 ID 6lu7_A pep YMHHMELPT aff 0.0826 aff_rescale 0.3506 cle 0.0297 tap -
82 ID 6lu7_A pep MQNCVLKLK aff 0.0631 aff_rescale 0.2677 cle 0.1853 tap [illegible]
89 ID 6lu7_A pep LKVDTANPK aff 0.0559 aff_rescale 0.2371 cle 0.3105 tap 0
94 ID 6lu7_A pep ANPKTPKYK aff 0.0517 aff_rescale 0.2197 cle 0.4933 tap 0
189 ID 6lu7_A pep QTAQAAGTD aff 0.0925 aff_rescale 0.3928 cle 0.0233 tap -
4 ID 6lu7_A pep RKMAFPSGK aff 0.0597 aff_rescale 0.2533 cle 0.1516 tap 0.
122 ID 6lu7_A pep PSGVYQCAM aff 0.0703 aff_rescale 0.2986 cle 0.1694 tap -
121 ID 6lu7_A pep SPSGVYQCA aff 0.0519 aff_rescale 0.2204 cle 0.8655 tap -[illegible]
20 ID 6lu7_A pep VTCGTTTLN aff 0.0842 aff_rescale 0.3575 cle 0.1114 tap -1.
154 ID 6lu7_A pep YDCVSFCYM aff 0.0640 aff_rescale 0.2717 cle 0.2884 tap -
144 ID 6lu7_A pep SCGSVGFNI aff 0.0626 aff_rescale 0.2656 cle 0.1383 tap 0.
32 ID 6lu7_A pep LDDVVYCPR aff 0.0548 aff_rescale 0.2325 cle 0.1237 tap 0
134 ID 6lu7_A pep FTIKGSFLN aff 0.0829 aff_rescale 0.3522 cle 0.0460 tap -1.[illegible]
51 ID 6lu7_A pep NPNYEDLLI aff 0.0560 aff_rescale 0.2377 cle 0.3186 tap 0.[illegible]
291 ID 6lu7_A pep FTPFDVVRQ aff 0.0687 aff_rescale 0.2917 cle 0.1581 tap -0
183 ID 6lu7_A pep GPFVDRQTA aff 0.0468 aff_rescale 0.1986 cle 0.9554 tap -[illegible]
62 ID 6lu7_A pep SNHNFLVQA aff 0.0606 aff_rescale 0.2573 cle 0.4079 tap -0
10 ID 6lu7_A pep SGKVEGCMV aff 0.0541 aff_rescale 0.2297 cle 0.4221 tap -[illegible]
298 ID 6lu7_A pep RQCSGVTFQ aff 0.0615 aff_rescale 0.2610 cle 0.1047 tap [illegible]
75 ID 6lu7_A pep LRVIGHSMQ aff 0.0490 aff_rescale 0.2081 cle 0.4968 tap 0.
123 ID 6lu7_A pep SGVYQCAMR aff 0.0498 aff_rescale 0.2115 cle 0.0690 tap
66 ID 6lu7_A pep FLVQAGNVQ aff 0.0643 aff_rescale 0.2728 cle 0.0472 tap [illegible]
34 ID 6lu7_A pep DVVYCPRHV aff 0.0522 aff_rescale 0.2217 cle 0.3717 tap [illegible]
27 ID 6lu7_A pep LNGLWLDDV aff 0.0594 aff_rescale 0.2522 cle 0.1852 tap [illegible]
252 ID 6lu7_A pep PLSAQTGIA aff 0.0627 aff_rescale 0.2664 cle 0.4012 tap -1.
285 ID 6lu7_A pep ALLEDEFTP aff 0.0586 aff_rescale 0.2490 cle 0.0566 tap 0.
45 ID 6lu7_A pep TSEDMLNPN aff 0.0803 aff_rescale 0.3411 cle 0.0282 tap -1
124 ID 6lu7_A pep GVYQCAMRP aff 0.0594 aff_rescale 0.2523 cle 0.0355 tap
96 ID 6lu7_A pep PKTPKYKFV aff 0.0438 aff_rescale 0.1859 cle 0.5762 tap -0.
167 ID 6lu7_A pep LPTGVHAGT aff 0.0513 aff_rescale 0.2177 cle 0.7573 tap -1
203 ID 6lu7_A pep NVLAWLYAA aff 0.0616 aff_rescale 0.2617 cle 0.1526 tap -
86 ID 6lu7_A pep VLKLKVDTA aff 0.0554 aff_rescale 0.2350 cle 0.3815 tap -0
120 ID 6lu7_A pep GSPSGVYQC aff 0.0568 aff_rescale 0.2414 cle 0.1800 tap [illegible]
288 ID 6lu7_A pep EDEFTPFDV aff 0.0513 aff_rescale 0.2176 cle 0.4200 tap -0.
17 ID 6lu7_A pep MVQVTCGTT aff 0.0672 aff_rescale 0.2853 cle 0.0501 tap -[illegible]
28 ID 6lu7_A pep NGLWLDDVV aff 0.0566 aff_rescale 0.2401 cle 0.0923 tap [illegible]
25 ID 6lu7_A pep TTLNGLWLD aff 0.0816 aff_rescale 0.3465 cle 0.0297 tap -1
78 ID 6lu7_A pep IGHSMQNCV aff 0.0535 aff_rescale 0.2273 cle 0.1435 tap 0
176 ID 6lu7_A pep DLEGNFYGP aff 0.0589 aff_rescale 0.2503 cle 0.1471 tap -[illegible]
236 ID 6lu7_A pep KYNYEPLTQ aff 0.0463 aff_rescale 0.1968 cle 0.3186 tap [illegible]
149 ID 6lu7_A pep GFNIDYDCV aff 0.0451 aff_rescale 0.1915 cle 0.3232 tap 0
16 ID 6lu7_A pep CMVQVTCGT aff 0.0649 aff_rescale 0.2754 cle 0.0334 tap -[illegible]

218 ID 6lu7_A pep WFLNRFTTT aff 0.0564 aff_rescale 0.2394 cle 0.2177 tap -0

70 ID 6lu7_A pep AGNVQLRVI aff 0.0493 aff_rescale 0.2094 cle 0.1847 tap 0.

243 ID 6lu7_A pep TQDHVDILG aff 0.0739 aff_rescale 0.3136 cle 0.0647 tap -1

191 ID 6lu7_A pep AQAAGTDTT aff 0.0633 aff_rescale 0.2689 cle 0.0237 tap -

235 ID 6lu7_A pep MKYNYEPLT aff 0.0600 aff_rescale 0.2546 cle 0.0470 tap -

273 ID 6lu7_A pep QNGMNGRTI aff 0.0523 aff_rescale 0.2221 cle 0.0767 tap

277 ID 6lu7_A pep NGRTILGSA aff 0.0527 aff_rescale 0.2239 cle 0.3810 tap -0.

258 ID 6lu7_A pep GIAVLDMCA aff 0.0580 aff_rescale 0.2462 cle 0.1917 tap -

181 ID 6lu7_A pep FYGPFVDRQ aff 0.0550 aff_rescale 0.2337 cle 0.0743 tap 0

160 ID 6lu7_A pep CYMHHMELP aff 0.0538 aff_rescale 0.2286 cle 0.0262 tap

77 ID 6lu7_A pep VIGHSMQNC aff 0.0552 aff_rescale 0.2342 cle 0.0228 tap 0

178 ID 6lu7_A pep EGNFYGPFV aff 0.0520 aff_rescale 0.2208 cle 0.2363 tap -0

18 ID 6lu7_A pep VQVTCGTTT aff 0.0615 aff_rescale 0.2611 cle 0.0462 tap -0

190 ID 6lu7_A pep TAQAAGTDT aff 0.0656 aff_rescale 0.2786 cle 0.0243 tap -

108 ID 6lu7_A pep PGQTFSVLA aff 0.0562 aff_rescale 0.2384 cle 0.4881 tap -1

37 ID 6lu7_A pep YCPRHVICT aff 0.0630 aff_rescale 0.2677 cle 0.0565 tap -0.

9 ID 6lu7_A pep PSGKVEGCM aff 0.0574 aff_rescale 0.2438 cle 0.1230 tap -0

72 ID 6lu7_A pep NVQLRVIGH aff 0.0606 aff_rescale 0.2574 cle 0.0333 tap -0.

257 ID 6lu7_A pep TGIAVLDMC aff 0.0587 aff_rescale 0.2492 cle 0.0237 tap -0

272 ID 6lu7_A pep LQNGMNGRT aff 0.0633 aff_rescale 0.2687 cle 0.0269 tap -

292 ID 6lu7_A pep TPFDVVRQC aff 0.0498 aff_rescale 0.2115 cle 0.2813 tap -0

88 ID 6lu7_A pep KLKVDTANP aff 0.0506 aff_rescale 0.2147 cle 0.0636 tap 0

85 ID 6lu7_A pep CVLKLKVDT aff 0.0601 aff_rescale 0.2551 cle 0.0331 tap -0

1 ID 6lu7_A pep SGFRKMAFP aff 0.0549 aff_rescale 0.2332 cle 0.0275 tap 0.

36 ID 6lu7_A pep VYCPRHVIC aff 0.0493 aff_rescale 0.2092 cle 0.0416 tap 0.

148 ID 6lu7_A pep VGFNIDYDC aff 0.0542 aff_rescale 0.2303 cle 0.0253 tap -0

202 ID 6lu7_A pep VNVLAWLYA aff 0.0595 aff_rescale 0.2528 cle 0.0371 tap -

275 ID 6lu7_A pep GMNGRTILG aff 0.0618 aff_rescale 0.2623 cle 0.2592 tap -1

193 ID 6lu7_A pep AAGTDTTIT aff 0.0617 aff_rescale 0.2619 cle 0.0276 tap -0.

284 ID 6lu7_A pep SALLEDEFT aff 0.0604 aff_rescale 0.2563 cle 0.0245 tap -0.

244 ID 6lu7_A pep QDHVDILGP aff 0.0542 aff_rescale 0.2300 cle 0.0414 tap -0

137 ID 6lu7_A pep KGSFLNGSC aff 0.0536 aff_rescale 0.2275 cle 0.0300 tap -0

11 ID 6lu7_A pep GKVEGCMVQ aff 0.0455 aff_rescale 0.1932 cle 0.2424 tap -

30 ID 6lu7_A pep LWLDDVVYC aff 0.0488 aff_rescale 0.2070 cle 0.0253 tap

76 ID 6lu7_A pep RVIGHSMQN aff 0.0593 aff_rescale 0.2517 cle 0.0792 tap -0

152 ID 6lu7_A pep IDYDCVSFC aff 0.0506 aff_rescale 0.2147 cle 0.0337 tap 0.

249 ID 6lu7_A pep ILGPLSAQT aff 0.0583 aff_rescale 0.2477 cle 0.1454 tap -0.

217 ID 6lu7_A pep RWFLNRFTT aff 0.0522 aff_rescale 0.2216 cle 0.0781 tap -0

109 ID 6lu7_A pep GQTFSVLAC aff 0.0529 aff_rescale 0.2248 cle 0.0626 tap -0

8 ID 6lu7_A pep FPSGKVEGC aff 0.0537 aff_rescale 0.2278 cle 0.0300 tap -0.

207 ID 6lu7_A pep WLYAAVING aff 0.0626 aff_rescale 0.2658 cle 0.0294 tap -

102 ID 6lu7_A pep KFVRIQPGQ aff 0.0476 aff_rescale 0.2019 cle 0.0349 tap 0.

14 ID 6lu7_A pep EGCMVQVTC aff 0.0548 aff_rescale 0.2326 cle 0.0285 tap -

247 ID 6lu7_A pep VDILGPLSA aff 0.0533 aff_rescale 0.2262 cle 0.1825 tap -0.

281 ID 6lu7_A pep ILGSALLED aff 0.0670 aff_rescale 0.2843 cle 0.1918 tap -1.

296 ID 6lu7_A pep VVRQCSGVT aff 0.0549 aff_rescale 0.2330 cle 0.0824 tap -

232 ID 6lu7_A pep LVAMKYNYE aff 0.0647 aff_rescale 0.2745 cle 0.0261 tap

184 ID 6lu7_A pep PFVDRQTAQ aff 0.0425 aff_rescale 0.1803 cle 0.3517 tap -

103 ID 6lu7_A pep FVRIQPGQT aff 0.0556 aff_rescale 0.2359 cle 0.0268 tap -0.

266 ID 6lu7_A pep ASLKELLQN aff 0.0626 aff_rescale 0.2660 cle 0.0272 tap -1

280 ID 6lu7_A pep TILGSALLE aff 0.0645 aff_rescale 0.2740 cle 0.0276 tap -1.

248 ID 6lu7_A pep DILGPLSAQ aff 0.0513 aff_rescale 0.2180 cle 0.0402 tap -0.

100 ID 6lu7_A pep KYKFVRIQP aff 0.0449 aff_rescale 0.1907 cle 0.0347 tap 0.

188 ID 6lu7_A pep RQTAQAAGT aff 0.0554 aff_rescale 0.2354 cle 0.0255 tap -

127 ID 6lu7_A pep QCAMRPNFT aff 0.0571 aff_rescale 0.2424 cle 0.0226 tap -

238 ID 6lu7_A pep NYEPLTQDH aff 0.0501 aff_rescale 0.2127 cle 0.1207 tap -

143 ID 6lu7_A pep GSCGSVGFN aff 0.0636 aff_rescale 0.2701 cle 0.0229 tap -1

155 ID 6lu7_A pep DCVSFCYMH aff 0.0541 aff_rescale 0.2297 cle 0.1132 tap -

57 ID 6lu7_A pep LLIRKSNHN aff 0.0611 aff_rescale 0.2593 cle 0.1002 tap -1.

7 ID 6lu7_A pep AFPSGKVEG aff 0.0551 aff_rescale 0.2341 cle 0.1583 tap -1.

91 ID 6lu7_A pep VDTANPKTP aff 0.0479 aff_rescale 0.2032 cle 0.0261 tap -0

171 ID 6lu7_A pep VHAGTDLEG aff 0.0610 aff_rescale 0.2590 cle 0.0517 tap -

182 ID 6lu7_A pep YGPFVDRQT aff 0.0577 aff_rescale 0.2452 cle 0.0597 tap -1

172 ID 6lu7_A pep HAGTDLEGN aff 0.0629 aff_rescale 0.2671 cle 0.0235 tap -

101 ID 6lu7_A pep YKFVRIQPG aff 0.0577 aff_rescale 0.2450 cle 0.0599 tap -1

135 ID 6lu7_A pep TIKGSFLNG aff 0.0554 aff_rescale 0.2354 cle 0.1691 tap -1.

99 ID 6lu7_A pep PKYKFVRIQ aff 0.0479 aff_rescale 0.2032 cle 0.0317 tap -0.

13 ID 6lu7_A pep VEGCMVQVT aff 0.0535 aff_rescale 0.2272 cle 0.0339 tap -

166 ID 6lu7_A pep ELPTGVHAG aff 0.0588 aff_rescale 0.2497 cle 0.1160 tap -1

26 ID 6lu7_A pep TLNGLWLDD aff 0.0656 aff_rescale 0.2784 cle 0.0332 tap -1

119 ID 6lu7_A pep NGSPSGVYQ aff 0.0470 aff_rescale 0.1996 cle 0.0459 tap -

112 ID 6lu7_A pep FSVLACYNG aff 0.0582 aff_rescale 0.2471 cle 0.0366 tap -1

87 ID 6lu7_A pep LKLKVDTAN aff 0.0562 aff_rescale 0.2385 cle 0.0272 tap -1

237 ID 6lu7_A pep YNYEPLTQD aff 0.0567 aff_rescale 0.2406 cle 0.1788 tap -1

21 ID 6lu7_A pep TCGTTTLNG aff 0.0599 aff_rescale 0.2544 cle 0.0236 tap -1.

56 ID 6lu7_A pep DLLIRKSNH aff 0.0515 aff_rescale 0.2187 cle 0.0788 tap -0.

116 ID 6lu7_A pep ACYNGSPSG aff 0.0529 aff_rescale 0.2246 cle 0.0434 tap -

43 ID 6lu7_A pep ICTSEDMLN aff 0.0564 aff_rescale 0.2396 cle 0.0232 tap -1.

216 ID 6lu7_A pep DRWFLNRFT aff 0.0478 aff_rescale 0.2031 cle 0.0510 tap -

105 ID 6lu7_A pep RIQPGQTFS aff 0.0615 aff_rescale 0.2613 cle 0.0430 tap -1.

269 ID 6lu7_A pep KELLQNGMN aff 0.0549 aff_rescale 0.2333 cle 0.0352 tap -

111 ID 6lu7_A pep TFSVLACYN aff 0.0546 aff_rescale 0.2318 cle 0.0248 tap -1

267 ID 6lu7_A pep SLKELLQNG aff 0.0532 aff_rescale 0.2261 cle 0.0444 tap -1

255 ID 6lu7_A pep AQTGIAVLD aff 0.0570 aff_rescale 0.2422 cle 0.0684 tap -1

206 ID 6lu7_A pep AWLYAAVIN aff 0.0502 aff_rescale 0.2132 cle 0.0224 tap -

33 ID 6lu7_A pep DDVVYCPRH aff 0.0478 aff_rescale 0.2031 cle 0.1436 tap -1

186 ID 6lu7_A pep VDRQTAQAA aff 0.0481 aff_rescale 0.2042 cle 0.0681 tap -

73 ID 6lu7_A pep VQLRVIGHS aff 0.0618 aff_rescale 0.2623 cle 0.0330 tap -1.

213 ID 6lu7_A pep INGDRWFLN aff 0.0568 aff_rescale 0.2413 cle 0.0270 tap -1
187 ID 6lu7_A pep DRQTAQAAG aff 0.0552 aff_rescale 0.2343 cle 0.0473 tap -
64 ID 6lu7_A pep HNFLVQAGN aff 0.0537 aff_rescale 0.2279 cle 0.0265 tap -1
221 ID 6lu7_A pep NRFTTTLND aff 0.0547 aff_rescale 0.2323 cle 0.0319 tap -1
270 ID 6lu7_A pep ELLQNGMNG aff 0.0543 aff_rescale 0.2307 cle 0.0469 tap -
113 ID 6lu7_A pep SVLACYNGS aff 0.0627 aff_rescale 0.2663 cle 0.0227 tap -2
170 ID 6lu7_A pep GVHAGTDLE aff 0.0557 aff_rescale 0.2364 cle 0.0257 tap -
54 ID 6lu7_A pep YEDLLIRKS aff 0.0655 aff_rescale 0.2783 cle 0.0318 tap -2.4
138 ID 6lu7_A pep GSFLNGSCG aff 0.0520 aff_rescale 0.2209 cle 0.0391 tap -1
48 ID 6lu7_A pep DMLNPNYED aff 0.0598 aff_rescale 0.2538 cle 0.0934 tap -2
158 ID 6lu7_A pep SFCYMHHME aff 0.0516 aff_rescale 0.2189 cle 0.0230 tap -
147 ID 6lu7_A pep SVGFNIDYD aff 0.0576 aff_rescale 0.2444 cle 0.0281 tap -1
3 ID 6lu7_A pep FRKMAFPSG aff 0.0505 aff_rescale 0.2146 cle 0.0527 tap -1.
115 ID 6lu7_A pep LACYNGSPS aff 0.0606 aff_rescale 0.2574 cle 0.0228 tap -2
250 ID 6lu7_A pep LGPLSAQTG aff 0.0537 aff_rescale 0.2281 cle 0.0595 tap -1
162 ID 6lu7_A pep MHHMELPTG aff 0.0511 aff_rescale 0.2171 cle 0.0284 tap -
125 ID 6lu7_A pep VYQCAMRPN aff 0.0471 aff_rescale 0.1999 cle 0.0244 tap -
230 ID 6lu7_A pep FNLVAMKYN aff 0.0543 aff_rescale 0.2306 cle 0.0248 tap -
276 ID 6lu7_A pep MNGRTILGS aff 0.0637 aff_rescale 0.2703 cle 0.0382 tap -2
208 ID 6lu7_A pep LYAAVINGD aff 0.0523 aff_rescale 0.2220 cle 0.0391 tap -1
262 ID 6lu7_A pep LDMCASLKE aff 0.0546 aff_rescale 0.2319 cle 0.0273 tap -
168 ID 6lu7_A pep PTGVHAGTD aff 0.0623 aff_rescale 0.2647 cle 0.0340 tap -
175 ID 6lu7_A pep TDLEGNFYG aff 0.0528 aff_rescale 0.2240 cle 0.0636 tap -1
141 ID 6lu7_A pep LNGSCGSVG aff 0.0547 aff_rescale 0.2322 cle 0.0238 tap -1
287 ID 6lu7_A pep LEDEFTPFD aff 0.0552 aff_rescale 0.2344 cle 0.0852 tap -2.
6 ID 6lu7_A pep MAFPSGKVE aff 0.0476 aff_rescale 0.2021 cle 0.0253 tap -1.
40 ID 6lu7_A pep RHVICTSED aff 0.0506 aff_rescale 0.2150 cle 0.0535 tap -1.
294 ID 6lu7_A pep FDVVRQCSG aff 0.0534 aff_rescale 0.2268 cle 0.0473 tap -
150 ID 6lu7_A pep FNIDYDCVS aff 0.0609 aff_rescale 0.2587 cle 0.0275 tap -2
220 ID 6lu7_A pep LNRFTTTLN aff 0.0493 aff_rescale 0.2095 cle 0.0405 tap -1
139 ID 6lu7_A pep SFLNGSCGS aff 0.0554 aff_rescale 0.2353 cle 0.0320 tap -2.
71 ID 6lu7_A pep GNVQLRVIG aff 0.0483 aff_rescale 0.2049 cle 0.0258 tap -1.
15 ID 6lu7_A pep GCMVQVTCG aff 0.0499 aff_rescale 0.2118 cle 0.0306 tap -
145 ID 6lu7_A pep CGSVGFNID aff 0.0541 aff_rescale 0.2298 cle 0.0319 tap -2
63 ID 6lu7_A pep NHNFLVQAG aff 0.0463 aff_rescale 0.1964 cle 0.0270 tap -1
259 ID 6lu7_A pep IAVLDMCAS aff 0.0548 aff_rescale 0.2325 cle 0.0246 tap -2
282 ID 6lu7_A pep LGSALLEDE aff 0.0507 aff_rescale 0.2152 cle 0.0228 tap -1
136 ID 6lu7_A pep IKGSFLNGS aff 0.0557 aff_rescale 0.2363 cle 0.0375 tap -2.
38 ID 6lu7_A pep CPRHVICTS aff 0.0504 aff_rescale 0.2141 cle 0.2237 tap -2.5
240 ID 6lu7_A pep EPLTQDHVD aff 0.0484 aff_rescale 0.2055 cle 0.1785 tap -2
84 ID 6lu7_A pep NCVLKLKVD aff 0.0462 aff_rescale 0.1962 cle 0.0269 tap -1
2 ID 6lu7_A pep GFRKMAFPS aff 0.0512 aff_rescale 0.2174 cle 0.0246 tap -2.
39 ID 6lu7_A pep PRHVICTSE aff 0.0447 aff_rescale 0.1899 cle 0.0785 tap -1.8
131 ID 6lu7_A pep RPNFTIKGS aff 0.0466 aff_rescale 0.1979 cle 0.1733 tap -2.

55 ID 6lu7_A pep EDLLIRKSN aff 0.0455 aff_rescale 0.1930 cle 0.0250 tap -1.[illegible]
47 ID 6lu7_A pep EDMLNPNYE aff 0.0456 aff_rescale 0.1935 cle 0.0259 tap -1[illegible]
179 ID 6lu7_A pep GNFYGPFVD aff 0.0437 aff_rescale 0.1856 cle 0.0480 tap -[illegible]
293 ID 6lu7_A pep PFDVVRQCS aff 0.0460 aff_rescale 0.1952 cle 0.0282 tap -2[illegible]

Number of MHC ligands 8 identified. Number of peptides 298. Protein name 6lu7_A

Rescale Binding Affinity	C-terminal Cleavage Affinity	TAP transport efficiency
3.3669	0.6229	2.702
2.6559	0.8852	2.957
1.3211	0.9565	2.857
1.1146	0.9725	2.998
0.8905	0.9722	2.706
0.7118	0.9755	2.723
0.6489	0.8406	2.676
0.4807	0.9503	2.568

2.7020 COMB 3.5954 <-E
2.9570 COMB 2.9365 <-E
.8570 COMB 1.6075 <-E
.9980 COMB 1.4104 <-E
2.7060 COMB 1.1717 <-E
.7230 COMB 0.9942 <-E
.6760 COMB 0.9088 <-E
5680 COMB 0.7517 <-E
0.5240 COMB 0.7377
2.9540 COMB 0.7345
2.5820 COMB 0.6969
0.2870 COMB 0.6905
.6410 COMB 0.6525
.2310 COMB 0.6311
.8460 COMB 0.6237
.3000 COMB 0.6223
.0740 COMB 0.6016
0.5120 COMB 0.5912
.5380 COMB 0.5794
2.8230 COMB 0.5711
.7600 COMB 0.5660
7290 COMB 0.5658
3.0780 COMB 0.5636
0.8130 COMB 0.5361
.4190 COMB 0.5331
2160 COMB 0.5268
.4610 COMB 0.5228
2.8180 COMB 0.5219
0.4490 COMB 0.5048
1170 COMB 0.5015

.0760 COMB 0.4960
7670 COMB 0.4929
5910 COMB 0.4921
.7880 COMB 0.4906
.6920 COMB 0.4887
.2640 COMB 0.4860
.3360 COMB 0.4792
.6130 COMB 0.4783
.9600 COMB 0.4709
.3490 COMB 0.4693
.5820 COMB 0.4648
0510 COMB 0.4638
.0470 COMB 0.4615
.6210 COMB 0.4605
0.6720 COMB 0.4598
.7710 COMB 0.4595
1.2640 COMB 0.4578
.0440 COMB 0.4564
.5620 COMB 0.4554
2.2380 COMB 0.4519
.1010 COMB 0.4499
4190 COMB 0.4494
.8460 COMB 0.4477
.4100 COMB 0.4435
7640 COMB 0.4422
.7720 COMB 0.4386
.1990 COMB 0.4337
5300 COMB 0.4314
2.6460 COMB 0.4307
0.3860 COMB 0.4300
.2850 COMB 0.4283
.0400 COMB 0.4271
.8100 COMB 0.4262
3100 COMB 0.4209
0.5540 COMB 0.4184
1.2650 COMB 0.4179
460 COMB 0.4154
.7710 COMB 0.4145
.2730 COMB 0.4103
300 COMB 0.4095
.1910 COMB 0.4056
7830 COMB 0.4053
.6480 COMB 0.4037
.7410 COMB 0.4036

0870 COMB 0.4034
.4330 COMB 0.4033
.2050 COMB 0.4031
.3320 COMB 0.4001
.5090 COMB 0.3997
0.0310 COMB 0.3987
1.1210 COMB 0.3904
.6750 COMB 0.3855
.9660 COMB 0.3843
9780 COMB 0.3832
.8170 COMB 0.3810
1310 COMB 0.3797
.7880 COMB 0.3794
5530 COMB 0.3718
1.3090 COMB 0.3710
.9000 COMB 0.3689
1.0880 COMB 0.3675
4770 COMB 0.3648
.6870 COMB 0.3629
0.1100 COMB 0.3616
8270 COMB 0.3607
.1290 COMB 0.3592
3080 COMB 0.3583
.1920 COMB 0.3580
1170 COMB 0.3544
.0800 COMB 0.3534
5130 COMB 0.3521
1.1070 COMB 0.3514
.2570 COMB 0.3502
.6000 COMB 0.3495
0.6290 COMB 0.3485
.0030 COMB 0.3482
.3530 COMB 0.3475
0.5810 COMB 0.3463
.5980 COMB 0.3456
0.3540 COMB 0.3449
0.3320 COMB 0.3413
0.0100 COMB 0.3404
.0800 COMB 0.3372
.2200 COMB 0.3334
.2680 COMB 0.3320
.0620 COMB 0.3314
.5140 COMB 0.3307
.5880 COMB 0.3302

.800 COMB 0.3301
6210 COMB 0.3297
-0.5810 COMB 0.3260
.5470 COMB 0.3229
.6970 COMB 0.3186
.4510 COMB 0.3162
1.6750 COMB 0.3126
7180 COMB 0.3120
-0.2440 COMB 0.3118
.8210 COMB 0.3092
.3150 COMB 0.3084
0.1530 COMB 0.3073
4150 COMB 0.3071
.9490 COMB 0.2985
2220 COMB 0.2980
210 COMB 0.2965
.4040 COMB 0.2952
.9590 COMB 0.2940
.5040 COMB 0.2933
.0460 COMB 0.2907
.2700 COMB 0.2902
1340 COMB 0.2893
1.2040 COMB 0.2820
.0380 COMB 0.2818
.0760 COMB 0.2812
.0130 COMB 0.2806
0300 COMB 0.2751
3400 COMB 0.2745
.4470 COMB 0.2730
0.3050 COMB 0.2729
.0110 COMB 0.2718
.2090 COMB 0.2708
-0.2950 COMB 0.2699
.4630 COMB 0.2691
.0020 COMB 0.2685
.2930 COMB 0.2660
.5590 COMB 0.2649
0.1510 COMB 0.2615
.8300 COMB 0.2594
.1480 COMB 0.2562
.3260 COMB 0.2560
.2080 COMB 0.2550
.2560 COMB 0.2528
.5770 COMB 0.2516

.4150	COMB	0.2513
2690	COMB	0.2506
.4690	COMB	0.2498
0.4590	COMB	0.2495
0.2680	COMB	0.2483
.2800	COMB	0.2476
.6800	COMB	0.2470
.5590	COMB	0.2470
.0420	COMB	0.2469
0.2880	COMB	0.2469
.1740	COMB	0.2463
.2020	COMB	0.2461
.4540	COMB	0.2453
0.7550	COMB	0.2445
.3630	COMB	0.2435
6730	COMB	0.2425
.4030	COMB	0.2421
.4220	COMB	0.2413
.2530	COMB	0.2401
-0.6720	COMB	0.2391
.3040	COMB	0.2385
.2770	COMB	0.2381
.4490	COMB	0.2376
0010	COMB	0.2374
3970	COMB	0.2353
.0240	COMB	0.2329
-0.5380	COMB	0.2315
1.4060	COMB	0.2309
.7210	COMB	0.2300
.6120	COMB	0.2294
.1500	COMB	0.2287
.1400	COMB	0.2250
0.1030	COMB	0.2245
.2530	COMB	0.2235
.8160	COMB	0.2228
0580	COMB	0.2227
9650	COMB	0.2212
.2420	COMB	0.2212
.2700	COMB	0.2207
2350	COMB	0.2206
1.0620	COMB	0.2171
.1950	COMB	0.2169
.4000	COMB	0.2169
7350	COMB	0.2168

9260 COMB 0.2167
0.5950 COMB 0.2156
-1.2650 COMB 0.2152
).3700 COMB 0.2146
.5120 COMB 0.2143
.1200 COMB 0.2140
3080 COMB 0.2127
2280 COMB 0.2126
.3290 COMB 0.2123
0.5610 COMB 0.2111
0.7070 COMB 0.2104
).4300 COMB 0.2093
1.3040 COMB 0.2084
0.8050 COMB 0.2065
3620 COMB 0.2062
0630 COMB 0.2047
.0760 COMB 0.2033
1.3390 COMB 0.1998
1.1120 COMB 0.1985
1.4700 COMB 0.1971
.1530 COMB 0.1963
3950 COMB 0.1910
3390 COMB 0.1910
0.8410 COMB 0.1902
1.5590 COMB 0.1891
1.9010 COMB 0.1883
).3910 COMB 0.1870
1.3620 COMB 0.1845
.1970 COMB 0.1827
1.7000 COMB 0.1824
.5120 COMB 0.1823
9810 COMB 0.1815
).9960 COMB 0.1813
2610 COMB 0.1801
).6730 COMB 0.1771
8580 COMB 0.1748
-1.2780 COMB 0.1747
.2220 COMB 0.1744
.1710 COMB 0.1742
.5780 COMB 0.1736
0.8590 COMB 0.1736
1.0330 COMB 0.1730
-0.8370 COMB 0.1726
9490 COMB 0.1698

1.5610 COMB 0.1673
-1.4900 COMB 0.1669
1.3060 COMB 0.1666
.4120 COMB 0.1665
-1.4610 COMB 0.1647
2.1410 COMB 0.1627
1.5660 COMB 0.1619
4360 COMB 0.1613
.3210 COMB 0.1608
2.1590 COMB 0.1599
-1.2890 COMB 0.1579
.8170 COMB 0.1578
3160 COMB 0.1567
2.1330 COMB 0.1541
.6640 COMB 0.1539
-1.3630 COMB 0.1532
-1.0510 COMB 0.1510
-1.6840 COMB 0.1501
.5440 COMB 0.1488
1.5860 COMB 0.1486
1.7570 COMB 0.1482
2.4320 COMB 0.1482
1.7360 COMB 0.1468
1.8120 COMB 0.1451
0750 COMB 0.1435
2530 COMB 0.1433
6310 COMB 0.1415
1.8650 COMB 0.1406
.4540 COMB 0.1401
.5200 COMB 0.1395
.0370 COMB 0.1382
.4950 COMB 0.1340
1.6560 COMB 0.1336
.0730 COMB 0.1309
1.3910 COMB 0.1309
2.1220 COMB 0.1301
.8230 COMB 0.1275
3260 COMB 0.1257
5940 COMB 0.1180
2.3580 COMB 0.1144
1.7290 COMB 0.1138
1540 COMB 0.1134
3160 COMB 0.1109
3640 COMB 0.1057

3610 COMB 0.1037
1.9580 COMB 0.0995
2.2020 COMB 0.0827
2.7240 COMB 0.0632

**Prediction score**
3.5954
2.9365
1.6075
1.4104
1.1717
0.9942
0.9088
0.7517
