# Supplementary material for: Immunoinformatics and Molecular Docking Studies Predicted Potential Multiepitope-Based Peptide Vaccine and Novel Compounds against Novel SARS-CoV-2 through Virtual Screening
Source: Biomed Res Int. 2021 Feb 26;2021:1596834. doi: 10.1155/2021/1596834 (PMC7910514; doi:10.1155/2021/1596834)
Supplement: Supplementary 3 — Physiochemical properties and population coverage analysis. [file 1596834.f3.pdf]

**Proteins**	**Molecular weight**	**Theorecical PI**	**Instability index**
Non-strutural	33796.64	5.95	2.65

Half-life	Stability profile
1.9 hours (mammalian reticulocytes, in vitro).	stable
>20 hours (yeast, in vivo).	
>10 hours (Escherichia coli, in vivo).	
