# Supplementary material for: Immunoinformatics and Molecular Docking Studies Predicted Potential Multiepitope-Based Peptide Vaccine and Novel Compounds against Novel SARS-CoV-2 through Virtual Screening
Source: Biomed Res Int. 2021 Feb 26;2021:1596834. doi: 10.1155/2021/1596834 (PMC7910514; doi:10.1155/2021/1596834)
Supplement: Supplementary 4 — Multiple sequence alignment. [file 1596834.f4.docx]

**Multiple Sequence Alignment**


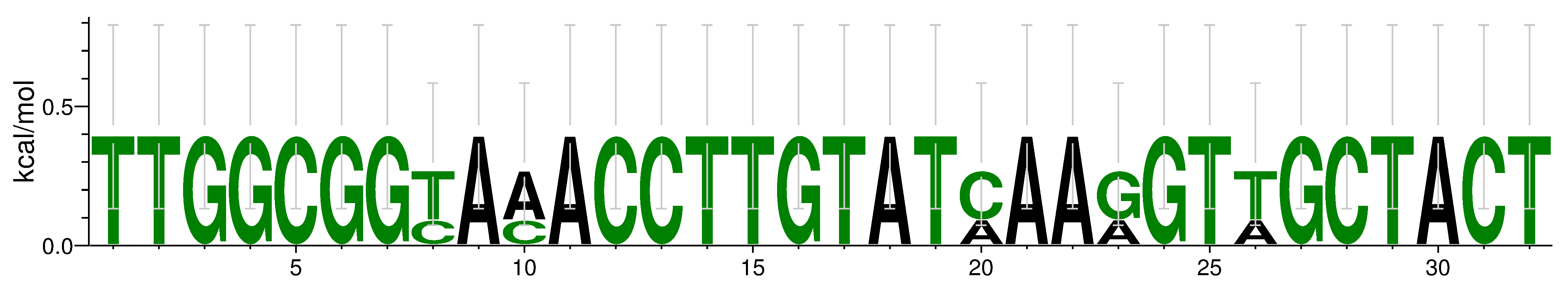
**Figure 1:** Highly conserved sequence among all three genomes.


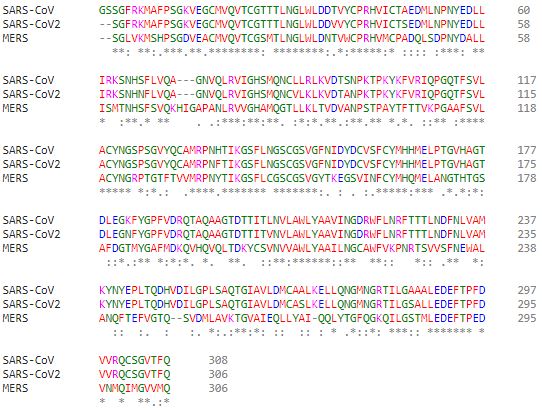


**Figure 2:** MSA of all three nonstructural proteins (6lu7 from SARS-CoV2, 4rsp from MERS and 3m3v from SARS-CoV) using Clustal Omega to check conserved domain sequence.
