# Supplementary material for: Immunoinformatics and Molecular Docking Studies Predicted Potential Multiepitope-Based Peptide Vaccine and Novel Compounds against Novel SARS-CoV-2 through Virtual Screening
Source: Biomed Res Int. 2021 Feb 26;2021:1596834. doi: 10.1155/2021/1596834 (PMC7910514; doi:10.1155/2021/1596834)

**Table 4:** FDA ligands selected by Molecular Docking studies, their properties evaluated by MOE, AutoDock, AutoDock Vina and AdmetSAR:

| **Ligands** | **Binding Energy**  **AD**  **(kcal/mol)** | **S score**  **MOE**  **(kcal/mol)** | **RMSD**  **Value** | **Molecular Weight**  **(g/mol)** | **A-logP**  **value** | **H-Bond Acceptor** | **H-Bond Donor** | **Rotatable Bond** | **Water solubility (logS)** | **Acute Oral Toxicity (kg/mol)** | **Interacting residues** | **Lipinski’s rule of Five violations** |
| --- | --- | --- | --- | --- | --- | --- | --- | --- | --- | --- | --- | --- |
| FDA-7 | -7.0 | -9.9153 | 1.7917 | 606.85 | 1.51 | 10 | 5 | 6 | -3.015 | 4.709 | VAL104  ARG105  ILE106  ASN151  PHE294 | 03 |
| FDA-378 | -7.9 | -9.4894 | 1.9330 | 367.58 | 3.02 | 5 | 5 | 9 | -2.598 | 2.964 | PHE8  VAL104  ARG105  GLN107  GLN110  ASN151  ILE152  ASP153  SER158  PHE294 | 00 |
| FDA-670 | -7.8 | -9.3083 | 1.8382 | 268.40 | 1.67 | 4 | 4 | 2 | -2.993 | 3.251 | VAL104  ARG105  GLN107  GLN110  ASN151  ASP153  PHE294 | 00 |
| FDA-592 | -7.6 | -8.6105 | 1.8580 | 265.44 | 2.57 | 3 | 3 | 0 | -2.579 | 3.738 | VAL104  ILE106  GLN110  ASN151  ASP153  SER158  PHE294 | 00 |
| FDA-636 | -7.0 | -8.5530 | 1.8580 | 476.63 | 1.34 | 7 | 7 | 6 | -2.442 | 4.249 | VAL123  GLY124  GLN127  LYS137  GLY170  VAL171  GLU290 | 01 |
| FDA-786 | -7.3 | -8.43 | 1.73 | 325.56 | 5.09 | 3 | 1 | 4 | -4.016 | 3.498 | VAL104  ARG105  ILE106  GLN107  GLN110  ASN151  ASP153  PHE294 | 02 |
| FDA-1232 | -7.0 | -8.0591 | 1.00 | 303.53 | 5.35 | 1 | 0 | 1 | -2.111 | 2.752 | GLN107  PRO108  GLY109  PRO132  PHE134  GLU240  HIS246 | 00 |
| FDA-899 | -7.0 | -7.91 | 1.46 | 322.49 | 2.40 | 4 | 2 | 2 | -2.141 | 2.758 | PHE8  VAL104  ARG105  ILE106  GLN107  GLN110  THR111 | 01 |
| FDA-839 | -7.1 | -7.8557 | 1.46 | 305.55 | 5.89 | 1 | 0 | 6 | -3.923 | 3.476 | VAL104  ILE106  GLN110  ASN151  ASP153  SER158  PHE294 |  |


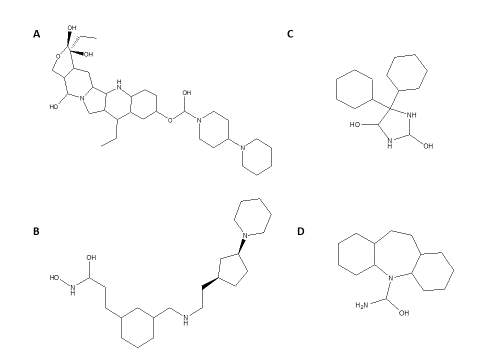

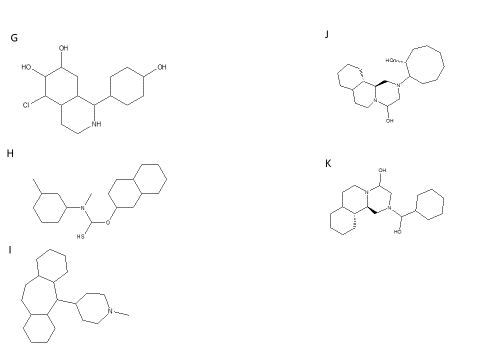

Supplement: Supplementary 5 — FDA ligands selected by molecular docking studies and their properties evaluated by MOE, AutoDock, AutoDock Vina, and admetSAR. [file 1596834.f5.docx]
